# Supplementary material for: Bayesian hierarchical mixture modelling to derive probabilistic iELISA thresholds for bovine brucellosis in endemic dairy systems
Source: PLoS One. 2026 Jul 30;21(7):e0347719. doi: 10.1371/journal.pone.0347719 (PMC13423031; doi:10.1371/journal.pone.0347719)
Supplement: S2 File — (DOC) [file pone.0347719.s002.doc]

Supplementary File 2. Stan code for the Bayesian Hierarchical Gaussian Mixture Model (HB-GMM) including likelihood definitions, priors, and non-centered parameterizations

// -----------------------------------------------------

// Three-population Hierarchical Gaussian Mixture Model

// - Healthy, Latent: Normal likelihood

// - Diseased: Student-t likelihood

// - Ordered population and herd means

// - Fully bounded non-centered parameterization

// - Hard caps to prevent Inf scales

// -----------------------------------------------------

data {

int<lower=1> N;

int<lower=1> N_herd;

array[N] int<lower=1, upper=N_herd> Herd;

vector[N] Log_SP;

vector<lower=0>[3] alpha;

}

parameters {

// ---------------- Population means ----------------

real mu_healthy;

real<lower=0> delta_latent;

real<lower=0> delta_diseased;

// ---------------- Observation SDs -----------------

real<lower=0.05, upper=1.0> sigma_healthy;

real<lower=0.05, upper=1.0> sigma_latent;

real<lower=0.05, upper=1.0> sigma_diseased;

// ---------------- Herd-level SD -------------------

real<lower=0, upper=0.25> sigma_herd;

// Non-centered herd effects (bounded)

matrix<lower=-1, upper=1>[N_herd, 3] z_herd;

// Herd-specific class prevalences

array[N_herd] simplex[3] P;

}

transformed parameters {

// ---------------- Ordered population means ----------------

real mu_latent = mu_healthy + delta_latent;

real mu_diseased = mu_latent + delta_diseased;

real nu_diseased = 4; // fixed degrees of freedom

// ---------------- Herd-specific means ----------------

matrix[N_herd, 3] lambda_herd;

for (j in 1:N_herd) {

real h = mu_healthy + sigma_herd * z_herd[j,1];

real l = mu_latent + sigma_herd * z_herd[j,2];

real d = mu_diseased + sigma_herd * z_herd[j,3];

// enforce ordering and hard bounds

lambda_herd[j,1] = fmin(fmax(h, -3), 3);

lambda_herd[j,2] = fmin(fmax(l, lambda_herd[j,1] + 0.05), 3);

lambda_herd[j,3] = fmin(fmax(d, lambda_herd[j,2] + 0.05), 3);

}

}

model {

// ---------------- Priors --------------------------

mu_healthy ~ normal(0, 1);

delta_latent ~ normal(0.6, 0.3);

delta_diseased ~ normal(1.0, 0.3);

sigma_healthy ~ normal(0.5, 0.2);

sigma_latent ~ normal(0.5, 0.2);

sigma_diseased ~ normal(0.6, 0.2);

sigma_herd ~ normal(0, 0.1);

to_vector(z_herd) ~ normal(0, 1);

for (j in 1:N_herd)

P[j] ~ dirichlet(alpha);

// ---------------- Mixture likelihood ----------------

for (i in 1:N) {

vector[3] lp;

lp[1] = log(P[Herd[i]][1]) + normal_lpdf(Log_SP[i] | lambda_herd[Herd[i],1], sigma_healthy);

lp[2] = log(P[Herd[i]][2]) + normal_lpdf(Log_SP[i] | lambda_herd[Herd[i],2], sigma_latent);

lp[3] = log(P[Herd[i]][3]) + student_t_lpdf(Log_SP[i] | nu_diseased,

lambda_herd[Herd[i],3],

sigma_diseased);

target += log_sum_exp(lp);

}

}

generated quantities {

real AUC_hl = Phi((mu_latent - mu_healthy) / sqrt(square(sigma_healthy) + square(sigma_latent)));

real AUC_ld = Phi((mu_diseased - mu_latent) / sqrt(square(sigma_latent) + square(sigma_diseased)));

real AUC_pop = Phi((mu_diseased - mu_healthy) / sqrt(square(sigma_healthy) + square(sigma_diseased)));

array[N] real y_rep;

for (i in 1:N) {

int comp = categorical_rng(P[Herd[i]]);

if (comp == 1)

y_rep[i] = normal_rng(lambda_herd[Herd[i],1], sigma_healthy);

else if (comp == 2)

y_rep[i] = normal_rng(lambda_herd[Herd[i],2], sigma_latent);

else

y_rep[i] = student_t_rng(nu_diseased, lambda_herd[Herd[i],3], sigma_diseased);

}

}
