# Supplementary material for: Bayesian hierarchical mixture modelling to derive probabilistic iELISA thresholds for bovine brucellosis in endemic dairy systems
Source: PLoS One. 2026 Jul 30;21(7):e0347719. doi: 10.1371/journal.pone.0347719 (PMC13423031; doi:10.1371/journal.pone.0347719)
Supplement: S3 File — (DOC) [file pone.0347719.s003.doc]

Supplementary File 2: R script for data preprocessing, model execution via ‘cmdstanr’, and posterior predictive checks

---

title: "Brucellosis in large dairy herds"

author: ""

output:

html_document: default

word_document: default

date: "`r format(Sys.Date(), '%B %d, %Y')`"

warning: no

---

```{r setup, include=FALSE}

knitr::opts_chunk$set(cache = FALSE)

```

```{r}

rm(list = ls())

gc()

# Note: Update path as needed for your local environment

setwd("")

library(readr)

brlh <- read.csv("Data.csv")

attach(brlh)

names(brlh)

```

```{r}

summary(SP)

```

```{r}

library(dplyr)

# Shift to avoid log(0) and negative values

c <- abs(min(brlh$SP, na.rm = TRUE)) + 1

brlh <- brlh %>%

mutate(Log_SP = log(SP + c))

glimpse(brlh)

```

```{r}

library(epiDisplay)

attach(brlh)

summ(Log_SP)

```

```{r}

library(ggplot2)

ggplot(brlh, aes(x = Log_SP)) +

geom_density(fill = "lightblue", alpha = 0.4) +

labs(

title = "Density of Log SP values",

x = "Log_SP",

y = "Density"

) +

theme_minimal(base_size = 13)

```

```{r}

ggplot(brlh, aes(x = Log_SP)) +

geom_histogram(aes(y = ..density..), bins = 50, fill = "lightgray", color = "black", alpha = 0.6) +

geom_density(color = "blue", linewidth = 1.2) +

labs(

title = "Histogram and Density of Log_SP",

x = "Log SP",

y = "Density"

) +

theme_minimal()

```

```{r}

library(ggplot2)

library(mclust)

library(dplyr)

library(viridis)

# ---- Data preparation ----

c_shift <- abs(min(brlh$SP, na.rm = TRUE)) + 1

brlh <- brlh %>%

mutate(Log_SP = log(SP + c_shift))

# ---- Fit 3-component GMM ----

gmm_sp <- Mclust(brlh$Log_SP)

summary(gmm_sp)

# ---- Extract parameters ----

means_sp <- gmm_sp$parameters$mean

sds_sp <- sqrt(gmm_sp$parameters$variance$sigmasq)

props_sp <- gmm_sp$parameters$pro

# ---- X-axis sequence ----

x_seq <- seq(min(brlh$Log_SP, na.rm = TRUE),

max(brlh$Log_SP, na.rm = TRUE),

length.out = 1000)

# ---- Component densities ----

dens_components <- sapply(seq_along(means_sp), function(k) {

props_sp[k] * dnorm(x_seq, mean = means_sp[k], sd = sds_sp[k])

})

# ---- Total mixture density ----

total_density <- rowSums(dens_components)

total_df <- data.frame(x = x_seq, density = total_density)

# ---- Long format for component densities ----

dens_df <- data.frame(

x = rep(x_seq, length(means_sp)),

density = as.vector(dens_components),

Component = factor(rep(paste0("Component ", seq_along(means_sp)), each = length(x_seq)))

)

# ---- Plot ----

ggplot() +

geom_histogram(

data = brlh,

aes(x = Log_SP, y = after_stat(density)),

bins = 30,

fill = "grey80",

color = "black",

alpha = 0.5

)+

# Observed density

geom_density(data = brlh, aes(x = Log_SP), color = "black", linewidth = 1) +

# GMM components (filled area)

geom_area(data = dens_df, aes(x = x, y = density, fill = Component),

alpha = 0.25) +

# GMM components (dashed lines)

geom_line(data = dens_df, aes(x = x, y = density, color = Component),

linetype = "dashed", linewidth = 1) +

# Total mixture density

geom_line(data = total_df, aes(x = x, y = density), color = "red", linewidth = 1.2) +

# Vertical lines for component means

geom_vline(xintercept = means_sp, linetype = "dotted", color = "black", linewidth = 1) +

# Annotate component means

geom_text(data = data.frame(x = means_sp, y = max(total_density) * 0.9,

label = round(means_sp, 2)),

aes(x = x, y = y, label = label),

angle = 90, vjust = -0.5, size = 4, color = "black") +

# Labels and theme

labs(

x = "Log(SP + shift)",

y = "Density",

fill = "GMM Component",

color = "GMM Component"

) +

scale_fill_viridis(discrete = TRUE, option = "D") +

scale_color_viridis(discrete = TRUE, option = "D") +

theme_minimal(base_size = 14) +

theme(

plot.title = element_text(face = "bold", hjust = 0.5),

legend.position = "top"

)

```

```{r}

# ---- Fit GMM (Automatic Component Selection) ----

# mclust now tests G = 1:9 and multiple covariance structures

gmm_sp <- Mclust(brlh$Log_SP)

# Extract the number of components selected

n_components <- gmm_sp$G

summary(gmm_sp)

# ---- Extract parameters ----

means_sp <- gmm_sp$parameters$mean

# Handle potential single component variance vs multiple

if(n_components > 1) {

sds_sp <- sqrt(gmm_sp$parameters$variance$sigmasq)

} else {

sds_sp <- sqrt(gmm_sp$parameters$variance$sigmasq)

}

props_sp <- gmm_sp$parameters$pro

# ---- X-axis sequence ----

x_seq <- seq(min(brlh$Log_SP, na.rm = TRUE),

max(brlh$Log_SP, na.rm = TRUE),

length.out = 1000)

# ---- Component densities (Dynamic) ----

dens_components <- sapply(seq_along(means_sp), function(k) {

props_sp[k] * dnorm(x_seq, mean = means_sp[k], sd = sds_sp[k])

})

# ---- Total mixture density ----

total_density <- if(n_components > 1) rowSums(dens_components) else dens_components

total_df <- data.frame(x = x_seq, density = total_density)

# ---- Long format for component densities ----

dens_df <- data.frame(

x = rep(x_seq, n_components),

density = as.vector(dens_components),

Component = factor(rep(paste0("Component ", seq_along(means_sp)), each = length(x_seq)))

)

# ---- Plot ----

ggplot() +

geom_histogram(

data = brlh,

aes(x = Log_SP, y = after_stat(density)),

bins = 30, fill = "grey80", color = "black", alpha = 0.5

) +

geom_density(data = brlh, aes(x = Log_SP), color = "black", linewidth = 1) +

# GMM components

geom_area(data = dens_df, aes(x = x, y = density, fill = Component), alpha = 0.25) +

geom_line(data = dens_df, aes(x = x, y = density, color = Component), linetype = "dashed") +

# Total mixture

geom_line(data = total_df, aes(x = x, y = density), color = "red", linewidth = 1.2) +

# Vertical lines for means

geom_vline(xintercept = means_sp, linetype = "dotted", color = "black") +

labs(

title = paste("GMM with", n_components, "Components (Optimal BIC)"),

x = "Log(SP + shift)",

y = "Density"

) +

scale_fill_viridis(discrete = TRUE) +

scale_color_viridis(discrete = TRUE) +

theme_minimal(base_size = 14) +

theme(legend.position = "top")

```

```{r}

## Extracting SD per “class”

for(k in 1:length(means_sp)) {

cat("Component", k,

"Mean =", round(means_sp[k], 3),

"SD =", round(sds_sp[k], 3),

"Proportion =", round(props_sp[k], 3), "\n")

}

```

```{r}

# -------------------------------

# 1. Libraries

# -------------------------------

library(cmdstanr)

library(dplyr)

library(tidyr)

library(ggplot2)

library(bayesplot)

# -------------------------------

# 2. Data preparation

# -------------------------------

# Shift SP to avoid log(0)

c_shift <- abs(min(brlh$SP, na.rm = TRUE)) + 1

brlh_clean <- brlh %>%

mutate(

Log_SP = log(SP + c_shift),

Herd_Idx = as.integer(factor(Herd)),

Log_SP_std = scale(Log_SP)[,1] # mean 0, SD 1

)

stan_data <- list(

N = nrow(brlh_clean),

N_herd = max(brlh_clean$Herd_Idx),

Herd = brlh_clean$Herd_Idx,

Log_SP = brlh_clean$Log_SP_std, # pass standardized data

alpha = c(2,2,2)

)

# -------------------------------

# 3. Initial Values Function

# -------------------------------

init_fun <- function() list(

mu_healthy = 0,

delta_latent = 0.5,

delta_diseased = 0.8,

sigma_healthy = 0.4,

sigma_latent = 0.5,

sigma_diseased = 0.5,

sigma_herd = 0.1,

z_herd = matrix(0, nrow=stan_data$N_herd, ncol=3),

P = matrix(rep(c(0.7,0.15,0.15), stan_data$N_herd),

nrow=stan_data$N_herd, byrow=TRUE)

)

# -------------------------------

# 4. Compile Stan model

# -------------------------------

# This tells Stan to put the 'binary' in a local temp folder

# while your .stan file stays in Google Drive.

mod <- cmdstan_model(

"Three_pop_BHGMM_model.stan",

dir = tempdir()

)

# -------------------------------

fit <- mod$sample(

data = stan_data,

chains = 4,

parallel_chains = 4,

iter_warmup = 2000,

iter_sampling = 6000,

adapt_delta = 0.99,

max_treedepth = 15,

init = init_fun

)

```

```{r}

library(bayesplot)

library(cmdstanr)

library(tidyverse)

# 1. Extract draws in matrix form

params_of_interest <- c("mu_healthy", "mu_latent", "mu_diseased",

"AUC_hl", "AUC_ld", "AUC_pop")

draws_matrix <- fit$draws(variables = params_of_interest, format = "matrix")

# 2. Traceplots

mcmc_trace(draws_matrix) +

ggtitle("Traceplots for key parameters")

# 3. Density plots

mcmc_dens(draws_matrix) +

ggtitle("Posterior densities for key parameters")

# 4. Optional: summary statistics

fit$summary(variables = params_of_interest) %>%

select(variable, mean, sd, rhat, ess_bulk, ess_tail) %>%

print()

```

```{r}

library(bayesplot)

library(cmdstanr)

library(tidyverse)

# -------------------------------

# 1. Extract posterior draws

# -------------------------------

params_of_interest <- c("mu_healthy", "mu_latent", "mu_diseased",

"AUC_hl", "AUC_ld", "AUC_pop")

draws_matrix <- fit$draws(variables = params_of_interest, format = "matrix")

# -------------------------------

# 2. Traceplots

# -------------------------------

mcmc_trace(draws_matrix) +

ggtitle("Traceplots for key parameters")

# -------------------------------

# 3. Density plots

# -------------------------------

mcmc_dens(draws_matrix) +

ggtitle("Posterior densities for key parameters")

# -------------------------------

# 4. Autocorrelation plots

# -------------------------------

mcmc_acf(draws_matrix, lags = 30) +

ggtitle("Autocorrelation of key parameters (first 30 lags)")

# -------------------------------

# 5. Optional: numeric summary

# -------------------------------

fit$summary(variables = params_of_interest) %>%

select(variable, mean, sd, rhat, ess_bulk, ess_tail) %>%

print()

# Clean theme for subsequent plots

theme_minimal() +

theme(panel.grid.minor = element_blank()) # Keeps it looking clean

```

```{r}

library(bayesplot)

library(ggplot2)

set.seed(123)

# Extract y_rep draws as a matrix: iterations × N

y_obs <- brlh_clean$Log_SP_std

y_rep <- fit$draws("y_rep", format = "matrix")

draw_idx <- sample(seq_len(nrow(y_rep)), size = 100)

y_rep_sub <- y_rep[draw_idx, ]

# -------------------------------

ppc_plot <- ppc_dens_overlay(

y = y_obs,

yrep = y_rep_sub

) +

labs(

) +

theme_minimal()

ppc_plot

# -------------------------------

# Save the plot as a tiff

ggsave(

"PPC_density_overlay.tiff",

plot = ppc_plot,

device = "tiff",

dpi = 700,

width = 8.5,

height = 6.5,

units = "in",

compression = "lzw"

)

```

```{r}

# -------------------------------

# 3. Posterior predictive intervals

# -------------------------------

# Show 50% and 90% predictive intervals

ppc_intervals(

y = y_obs,

yrep = y_rep_sub,

prob = 0.5

) +

labs(title = "PPC: 50% Posterior Predictive Intervals") +

theme_minimal()

ppc_intervals(

y = y_obs,

yrep = y_rep_sub,

prob = 0.9

) +

labs(title = "PPC: 90% Posterior Predictive Intervals") +

theme_minimal()

# -------------------------------

# 4. Component-wise predictive overlay (approximate)

# -------------------------------

# Visualize each mixture component:

# extract lambda_herd from draws (subset 50 draws)

lambda_draws <- fit$draws(variables = c("lambda_herd[1,1]", "lambda_herd[1,2]", "lambda_herd[1,3]"), format = "matrix")

# Example: plot predicted distributions for first herd

n_comp_draws <- 50

lambda_sample <- lambda_draws[1:n_comp_draws, ]

# Convert to long format for ggplot

lambda_long <- as.data.frame(lambda_sample) %>%

pivot_longer(cols = everything(), names_to = "component", values_to = "lambda")

# Plot density per component

ggplot(lambda_long, aes(x = lambda, fill = component)) +

geom_density(alpha = 0.4) +

labs(title = "Approximate Posterior Predictive Distribution by Component (Herd 1)",

x = "Log-SP", y = "Density") +

theme_minimal() +

scale_fill_brewer(palette = "Set1")

```

```{r}

# =========================================================

# Bayesian 3-Component Mixture: Reproducible Cutoffs

# =========================================================

library(cmdstanr)

library(dplyr)

library(tidyr)

library(ggplot2)

set.seed(20269)

# =========================================================

# 1. SCALE LOG-SP DATA (consistent with model fitting)

# =========================================================

log_sp_scaled <- scale(brlh_clean$Log_SP)

mu_log_std <- attr(log_sp_scaled, "scaled:center")

sd_log_std <- attr(log_sp_scaled, "scaled:scale")

c_val <- if(exists("c_shift")) as.numeric(c_shift) else 0

# Standardized log-SP for plotting (optional)

brlh_clean$Log_SP_std <- as.numeric(log_sp_scaled)

# =========================================================

# 2. STABLE BAYESIAN CUTOFF FUNCTION

# =========================================================

# Solve f1(x) = f2(x) for two normals

bayes_cutoff <- function(mu1, sd1, mu2, sd2) {

if (any(!is.finite(c(mu1, sd1, mu2, sd2)))) return(NA_real_)

# Quadratic coefficients

A <- 1/(2*sd1^2) - 1/(2*sd2^2)

B <- mu2/(sd2^2) - mu1/(sd1^2)

C <- mu1^2/(2*sd1^2) - mu2^2/(2*sd2^2) + log(sd2/sd1)

# Handle near-equal SDs

if (abs(A) < 1e-8) return((mu1 + mu2)/2)

disc <- B^2 - 4*A*C

if (disc < 0) return((mu1 + mu2)/2) # fallback

roots <- (-B + c(-1, 1) * sqrt(disc)) / (2*A)

# Pick root between the means

roots[roots > min(mu1, mu2) & roots < max(mu1, mu2)][1]

}

v_cutoff <- Vectorize(bayes_cutoff)

# =========================================================

# 3. EXTRACT POSTERIOR DRAWS

# =========================================================

post <- fit$draws(format = "df")

m_h <- post$mu_healthy

s_h <- post$sigma_healthy

m_l <- post$mu_latent

s_l <- post$sigma_latent

m_d <- post$mu_diseased

s_d <- post$sigma_diseased

# Diseased df (fixed or posterior mean)

nu_d <- if("nu_diseased" %in% colnames(post)) mean(post$nu_diseased, na.rm=TRUE) else 4

# =========================================================

# 4. CALCULATE POSTERIOR CUTOFF DISTRIBUTIONS

# =========================================================

cut_hl <- v_cutoff(m_h, s_h, m_l, s_l)

cut_ld <- v_cutoff(m_l, s_l, m_d, s_d)

# Median & 95% CI (posterior)

z_hl <- median(cut_hl, na.rm=TRUE)

z_ld <- median(cut_ld, na.rm=TRUE)

ci_hl <- quantile(cut_hl, c(0.025, 0.5, 0.975), na.rm=TRUE)

ci_ld <- quantile(cut_ld, c(0.025, 0.5, 0.975), na.rm=TRUE)

# =========================================================

# 5. BACK-TRANSFORM TO ORIGINAL SP SCALE

# =========================================================

final_sp_hl <- exp(z_hl * sd_log_std + mu_log_std) - c_val

final_sp_ld <- exp(z_ld * sd_log_std + mu_log_std) - c_val

ci_sp_hl <- exp(ci_hl * sd_log_std + mu_log_std) - c_val

ci_sp_ld <- exp(ci_ld * sd_log_std + mu_log_std) - c_val

# =========================================================

# 6. PREPARE DENSITY DATA (posterior means)

# =========================================================

x_seq <- seq(min(brlh_clean$Log_SP_std, na.rm=TRUE),

max(brlh_clean$Log_SP_std, na.rm=TRUE),

length.out = 2000)

dens_df <- tibble(

x = x_seq,

Healthy = dnorm(x, mean(m_h), mean(s_h)),

Latent = dnorm(x, mean(m_l), mean(s_l)),

Diseased = dt((x - mean(m_d))/mean(s_d), df=nu_d)/mean(s_d)

) %>% pivot_longer(-x, names_to="Class", values_to="Density")

# =========================================================

# 7. PUBLICATION-READY PLOT WITH OBSERVED HISTOGRAM

# =========================================================

final_plot <- ggplot() +

# Observed histogram (density scale)

geom_histogram(

data = brlh_clean,

aes(x = Log_SP_std, y = after_stat(density)),

bins = 50,

fill = "grey80",

color = "grey40",

alpha = 0.6

) +

# Posterior-mean component densities

geom_line(

data = dens_df,

aes(x = x, y = Density, color = Class),

linewidth = 1.1

) +

# Bayesian cutoffs

geom_vline(xintercept = c(z_hl, z_ld),

linetype = "dashed",

color = "grey30") +

# Cutoff labels

annotate("text", x = z_hl, y = max(dens_df$Density) * 0.9,

label = sprintf("H–L: %.2f SP", final_sp_hl),

angle = 90, vjust = -0.4, size = 3.5) +

annotate("text", x = z_ld, y = max(dens_df$Density) * 0.9,

label = sprintf("L–D: %.2f SP", final_sp_ld),

angle = 90, vjust = 1.4, size = 3.5) +

# Dual x-axis (standardized original SP)

scale_x_continuous(

name = "Standardized log-SP",

sec.axis = sec_axis(~ exp(. * sd_log_std + mu_log_std) - c_val,

name = "Original SP")

) +

scale_color_manual(values = c(

Healthy = "#1B9E77",

Latent = "#D95F02",

Diseased = "#7570B3"

)) +

theme_classic(base_size = 12) +

labs(y = "Density", color = "Population")

final_plot

# =========================================================

# 8. SAVE FIGURE

# =========================================================

ggsave("Bayesian_3Component_Density.tiff", plot=final_plot,

width=9, height=7, dpi=700, compression="lzw")

# =========================================================

# 9. PRINT RESULTS

# =========================================================

cat("\n--- FINAL POPULATION CUTOFFS ---\n")

cat(sprintf("Healthy–Latent cutoff: %.2f SP (95%% CI: %.2f–%.2f)\n",

final_sp_hl, ci_sp_hl[1], ci_sp_hl[3]))

cat(sprintf("Latent–Diseased cutoff: %.2f SP (95%% CI: %.2f–%.2f)\n",

final_sp_ld, ci_sp_ld[1], ci_sp_ld[3]))

```

```{r}

library(dplyr)

library(tidyr)

library(ggplot2)

library(posterior)

# =========================================================

# 1. Extract posterior draws of herd-specific proportions P

# =========================================================

draws_P <- fit$draws("P", format = "df")

# =========================================================

# 2. Compute population-level prevalence for each class

# =========================================================

prevalence_summary <- draws_P %>%

pivot_longer(

cols = starts_with("P["),

names_to = "parameter",

values_to = "proportion"

) %>%

# Extract class index from "P[herd,class]"

mutate(class_idx = as.numeric(gsub("P\\[\\d+,(\\d+)\\]", "\\1", parameter))) %>%

# Step A: Average across herds per draw

group_by(.draw, class_idx) %>%

summarise(avg_prevalence = mean(proportion), .groups = "drop") %>%

# Step B: Summarize posterior distribution

group_by(class_idx) %>%

summarise(

mean_prevalence = mean(avg_prevalence),

median_prevalence = median(avg_prevalence),

lower_95_CrI = quantile(avg_prevalence, 0.025),

upper_95_CrI = quantile(avg_prevalence, 0.975),

.groups = "drop"

) %>%

# Add class labels

mutate(

Class = factor(class_idx, levels = 1:3, labels = c("Healthy", "Latent", "Diseased"))

) %>%

select(Class, mean_prevalence, lower_95_CrI, upper_95_CrI)

# Print table

cat("\n--- Population-Level Class Prevalence (95% CrI) ---\n")

print(prevalence_summary)

# =========================================================

# 3. Prepare long-format data for plotting

# =========================================================

draws_P_long <- draws_P %>%

pivot_longer(cols = starts_with("P["), names_to = "parameter", values_to = "val") %>%

mutate(class_idx = as.numeric(gsub("P\\[\\d+,(\\d+)\\]", "\\1", parameter))) %>%

group_by(.draw, class_idx) %>%

summarise(avg_prev = mean(val), .groups = "drop") %>%

mutate(Class = factor(class_idx, levels = 1:3, labels = c("Healthy", "Latent", "Diseased")))

# =========================================================

# 4. Create publication-ready density plot

# =========================================================

prevalence_plot <- ggplot(draws_P_long, aes(x = avg_prev, fill = Class, color = Class)) +

geom_density(alpha = 0.5, linewidth = 0.7) +

theme_classic(base_size = 12) +

scale_fill_manual(values = c("Healthy" = "#1B9E77", "Latent" = "#D95F02", "Diseased" = "#7570B3")) +

scale_color_manual(values = c("Healthy" = "#1B9E77", "Latent" = "#D95F02", "Diseased" = "#7570B3")) +

labs(

x = "Average Proportion",

y = "Density",

fill = "Class",

color = "Class"

) +

geom_vline(data = prevalence_summary,

aes(xintercept = mean_prevalence, color = Class),

linetype = "dashed", linewidth = 0.8)

# Show the plot

print(prevalence_plot)

# =========================================================

# 5. Save high-resolution TIFF

# =========================================================

ggsave(

filename = "Posterior_Population_Prevalence.tiff",

plot = prevalence_plot,

width = 9,

height = 7,

dpi = 700,

compression = "lzw"

)

```

```{r}

library(dplyr)

# =========================================================

# 1. EXTRACT POSTERIOR DRAWS

# =========================================================

post <- fit$draws(format = "df")

mu_mat <- data.frame(

Healthy = post$mu_healthy,

Latent = post$mu_latent,

Diseased = post$mu_diseased

)

# =========================================================

# 2. SUMMARY FUNCTION

# =========================================================

summarise_post <- function(x) {

c(

mean(x, na.rm = TRUE),

quantile(x, 0.025, na.rm = TRUE),

quantile(x, 0.975, na.rm = TRUE)

)

}

# =========================================================

# 3. CREATE SUMMARY DATA FRAME

# =========================================================

log_std_summary <- as.data.frame(

t(sapply(mu_mat, summarise_post))

)

colnames(log_std_summary) <- c("Mean", "Lo95", "Hi95")

log_std_summary$Class <- rownames(log_std_summary)

rownames(log_std_summary) <- NULL

log_std_summary <- log_std_summary %>%

select(Class, Mean, Lo95, Hi95)

# =========================================================

# 4. BACK-TRANSFORM TO ORIGINAL SP SCALE

# =========================================================

sp_summary <- log_std_summary %>%

mutate(

Mean_SP = exp(Mean * sd_log_std + mu_log_std) - c_shift,

Lo95_SP = exp(Lo95 * sd_log_std + mu_log_std) - c_shift,

Hi95_SP = exp(Hi95 * sd_log_std + mu_log_std) - c_shift

)

# View results

sp_summary

```

```{r}

#Weighted Prevalence

# -----------------------------------

# Extract all posterior draws as a data frame

post <- fit$draws(format = "df")

# -----------------------------------

# Herd-size weights

# -----------------------------------

herd_counts <- table(brlh$Herd)

herd_weights <- as.numeric(herd_counts) / sum(herd_counts)

# Number of herds

n_herd <- length(herd_weights)

# -----------------------------------

# Identify prevalence columns by class

# -----------------------------------

idx_ph <- grep("^P\\[.*,1\\]$", colnames(post)) # Healthy

idx_pl <- grep("^P\\[.*,2\\]$", colnames(post)) # Latent

idx_pd <- grep("^P\\[.*,3\\]$", colnames(post)) # Diseased

# -----------------------------------

# Herd-size–weighted prevalence per draw

# -----------------------------------

prev_healthy_draws <- as.numeric(

as.matrix(post[, idx_ph]) %*% herd_weights

)

prev_latent_draws <- as.numeric(

as.matrix(post[, idx_pl]) %*% herd_weights

)

prev_diseased_draws <- as.numeric(

as.matrix(post[, idx_pd]) %*% herd_weights

)

# -----------------------------------

# Summarise with 95% credible intervals

# -----------------------------------

overall_prev_weighted <- data.frame(

Class = c("Healthy", "Latent", "Diseased"),

Mean = c(mean(prev_healthy_draws),

mean(prev_latent_draws),

mean(prev_diseased_draws)),

Lo95 = c(quantile(prev_healthy_draws, 0.025),

quantile(prev_latent_draws, 0.025),

quantile(prev_diseased_draws, 0.025)),

Hi95 = c(quantile(prev_healthy_draws, 0.975),

quantile(prev_latent_draws, 0.975),

quantile(prev_diseased_draws, 0.975))

)

library(dplyr)

overall_prev_weighted <- overall_prev_weighted %>%

mutate(

Class = factor(Class, levels = c("Healthy", "Latent", "Diseased")),

Label = paste0(round(Mean*100,1), "% [",

round(Lo95*100,1), "-",

round(Hi95*100,1), "%]")

)

print(overall_prev_weighted)

```

```{r}

# =========================================================

# Polished Herd-Specific Cutoffs (C2) - Anonymous

# =========================================================

library(dplyr)

library(tidyr)

library(ggplot2)

# ---------------------------

# 1. Setup & Anonymization

# ---------------------------

# Force dplyr to avoid conflicts with plyr

herd_mapping <- brlh %>%

dplyr::group_by(Herd) %>%

dplyr::summarise(n = dplyr::n(), .groups = "drop") %>%

dplyr::arrange(Herd) %>%

dplyr::mutate(

Herd_Number = row_number(),

Anon_Label = paste0("Herd ", Herd_Number, " (n=", n, ")")

)

post <- fit$draws(format = "df")

n_samples <- nrow(post)

herd_ids <- herd_mapping$Herd_Number

# Pre-requisite scaling parameters

log_values <- log(brlh$SP + c_shift)

mean_log_sp <- mean(log_values, na.rm = TRUE)

sd_log_sp <- sd(log_values, na.rm = TRUE)

# ---------------------------

# 2. Herd-Specific Cutoff Calculation

# ---------------------------

dstent <- function(x, mu, sigma, nu = 4) {

return(dt((x - mu) / sigma, df = nu) / sigma)

}

herd_results_c2 <- vector("list", length(herd_ids))

for (h in herd_ids) {

cuts_standardized <- numeric(n_samples)

# Extract herd-specific column names

col_l <- paste0("lambda_herd[", h, ",2]")

col_d <- paste0("lambda_herd[", h, ",3]")

col_pl <- paste0("P[", h, ",2]")

col_pd <- paste0("P[", h, ",3]")

for (i in seq_len(n_samples)) {

m_l <- post[[col_l]][i]

m_d <- post[[col_d]][i]

s_l <- post$sigma_latent[i]

s_d <- post$sigma_diseased[i]

p_l <- post[[col_pl]][i]

p_d <- post[[col_pd]][i]

f_diff <- function(x) (p_l * dnorm(x, m_l, s_l)) - (p_d * dstent(x, m_d, s_d, nu = 4))

cuts_standardized[i] <- tryCatch(

uniroot(f_diff, lower = m_l, upper = m_d)$root,

error = function(e) NA_real_

)

}

# Back-transformation

valid_cuts <- cuts_standardized[!is.na(cuts_standardized)]

cuts_sp <- exp(valid_cuts * sd_log_sp + mean_log_sp) - c_shift

herd_results_c2[[h]] <- tibble(

Herd_Number = h,

Mean_C2 = mean(cuts_sp, na.rm = TRUE),

Lower_C2 = quantile(cuts_sp, 0.025, na.rm = TRUE),

Upper_C2 = quantile(cuts_sp, 0.975, na.rm = TRUE)

)

}

c2_data <- bind_rows(herd_results_c2) %>%

left_join(herd_mapping, by = "Herd_Number")

# ---------------------------------------------------------

# 3. Publication Quality Forest Plot

# ---------------------------------------------------------

# Global reference value

global_cutoff_val <- 82.00

c2_plot_polished <- ggplot(c2_data, aes(y = reorder(Anon_Label, Mean_C2), x = Mean_C2)) +

# Reference line for global cutoff

geom_vline(xintercept = global_cutoff_val, linetype = "dashed", color = "red", linewidth = 0.8) +

# 95% Credible Intervals

geom_errorbarh(aes(xmin = Lower_C2, xmax = Upper_C2),

height = 0.3, color = "#56B4E9", linewidth = 0.8) +

# Mean points

geom_point(size = 3, color = "#004E82") +

# Value labels

geom_text(aes(label = sprintf("%.1f", Mean_C2)),

vjust = -1.2, size = 3.5, fontface = "bold") +

# Global Cutoff Annotation

annotate("label", x = global_cutoff_val, y = 1, label = paste0("Global Cutoff: ", global_cutoff_val),

color = "red", fill = "white", size = 3.5, fontface = "bold", label.size = 0.4) +

labs(

x = "SP Value Cutoff (Latent vs Diseased)",

y = "Anonymized Herd (Sample Size)"

) +

theme_classic(base_size = 14) +

theme(

axis.text.y = element_text(color = "black"),

axis.text.x = element_text(color = "black"),

plot.margin = margin(10, 20, 10, 10)

)

print(c2_plot_polished)

# Reorder columns for clarity using the anonymized label

herd_c2_table <- c2_data %>%

select(Herd_Number, Anon_Label, Mean_C2, Lower_C2, Upper_C2) %>%

arrange(Herd_Number)

# Print table

print(herd_c2_table)

# ---------------------------

# 4. Save High-Res

# ---------------------------

ggsave(

filename = "Herd_specific_C2_Cutoffs.tiff",

plot = c2_plot_polished,

device = "tiff",

dpi = 700,

width = 9,

height = 9,

compression = "lzw"

)

```

```{r}

# =========================================================

# Herd-Level True Prevalence (Posterior P[herd,3])

# =========================================================

library(dplyr)

library(tidyr)

library(ggplot2)

library(scales)

# ---------------------------

# 1. Herd IDs and weights

# ---------------------------

herd_counts <- table(brlh$Herd)

herd_weights <- as.numeric(herd_counts) / sum(herd_counts)

herd_ids <- seq_along(herd_counts)

n_samples <- nrow(post)

# ---------------------------

# 2. Extract posterior draws for True Prevalence

# ---------------------------

herd_true_prev <- matrix(NA, nrow = n_samples, ncol = length(herd_ids))

for (h in herd_ids) {

herd_true_prev[, h] <- post[[paste0("P[", h, ",3]")]]

}

# ---------------------------

# 3. Summarise per herd

# ---------------------------

herd_true_summary <- data.frame(

Herd_Number = herd_ids,

Mean_Prev = colMeans(herd_true_prev),

Lo95 = apply(herd_true_prev, 2, quantile, 0.025),

Hi95 = apply(herd_true_prev, 2, quantile, 0.975)

) %>%

left_join(herd_mapping %>% select(Herd_Number, Anon_Label), by = "Herd_Number") %>%

select(Herd_Number, Anon_Label, Mean_Prev, Lo95, Hi95)

# ---------------------------

# 4. Weighted Overall True Prevalence

# ---------------------------

overall_true_prev_draws <- herd_true_prev %*% herd_weights

overall_true_prev <- mean(overall_true_prev_draws)

overall_lo <- quantile(overall_true_prev_draws, 0.025)

overall_hi <- quantile(overall_true_prev_draws, 0.975)

cat(sprintf("\nWeighted True Prevalence: %.2f%% (95%% CI %.2f–%.2f%%)\n",

overall_true_prev*100, overall_lo*100, overall_hi*100))

# ---------------------------

# 5. Forest Plot

# ---------------------------

true_prev_plot <- ggplot(herd_true_summary, aes(y = reorder(Anon_Label, Mean_Prev), x = Mean_Prev)) +

# 95% Credible Intervals

geom_errorbarh(aes(xmin = Lo95, xmax = Hi95), height = 0.3, color = "#004E82", size = 0.8) +

# Posterior Mean

geom_point(size = 3.5, color = "#56B4E9") +

# Overall True Prevalence

geom_vline(xintercept = overall_true_prev, linetype = "dashed", color = "red", size = 1) +

# Labels for Means

geom_text(aes(label = sprintf("%.1f%%", Mean_Prev*100)),

hjust = -0.5, vjust = -0.3, size = 3.5, fontface = "bold", color = "black") +

# Annotation for Overall

annotate("text", x = overall_true_prev, y = 1,

label = paste0("Weighted Overall: ", round(overall_true_prev*100, 2), "%"),

color = "red", vjust = -1.5, hjust = -0.1, fontface = "bold") +

scale_x_continuous(labels = scales::percent_format(accuracy = 1),

expand = expansion(mult = c(0, 0.2))) +

labs(

x = "True Prevalence (%)",

y = NULL,

subtitle = "Posterior mean and 95% credible intervals from Bayesian LCM"

) +

theme_classic(base_size = 14) +

theme(

axis.text = element_text(color = "black"),

plot.title = element_text(face = "bold"),

plot.subtitle = element_text(color = "grey40")

)

print(true_prev_plot)

# ---------------------------

# 6. Final Table

# ---------------------------

final_true_table <- herd_true_summary %>%

mutate(`True Prevalence (95% CI)` = sprintf("%.2f%% (%.2f–%.2f%%)", Mean_Prev*100, Lo95*100, Hi95*100)) %>%

select(Herd = Anon_Label, `True Prevalence (95% CI)`)

# Add overall row

final_true_table <- bind_rows(

final_true_table,

tibble(Herd = "WEIGHTED OVERALL",

`True Prevalence (95% CI)` = sprintf("%.2f%% (%.2f–%.2f%%)",

overall_true_prev*100, overall_lo*100, overall_hi*100))

)

print(final_true_table)

# ---------------------------

# 7. Save Plot

# ---------------------------

ggsave("Herd_Level_True_Prevalence.tiff", plot = true_prev_plot,

width = 10, height = 8, dpi = 700, compression = "lzw", bg = "white")

```

```{r}

saveRDS(fit, "BGHMM_fit_full_posterior.rds")

file.exists("BGHMM_fit_full_posterior.rds")

```

```{r}

fit$save_output_files(dir = ".", basename = "BGHMM_cmdstan")

```

```{r}

fit <- readRDS("BGHMM_fit_full_posterior.rds")

post <- fit$draws(format = "df")

```

```{r}

# =========================================================

# Diagnostic Performance Metrics (Automated)

# =========================================================

library(dplyr)

library(tidyr)

library(tibble)

# ---------------------------------------------------------

# 1. Automate Cutoff Retrieval

# ---------------------------------------------------------

# Ensure these variables exist from your previous "Cutoff" script

# If not, fallback to the hardcoded values (safety check)

val_c1 <- if(exists("final_sp_hl")) final_sp_hl else 10.71

val_c2 <- if(exists("final_sp_ld")) final_sp_ld else 82.21

# Standardize these cutoffs using the same scaling logic as the model

cutoff1_std <- (log(val_c1 + c_shift) - mu_log_std) / sd_log_std

cutoff2_std <- (log(val_c2 + c_shift) - mu_log_std) / sd_log_std

```
