# Supplementary material for: Bayesian hierarchical mixture modelling to derive probabilistic iELISA thresholds for bovine brucellosis in endemic dairy systems
Source: PLoS One. 2026 Jul 30;21(7):e0347719. doi: 10.1371/journal.pone.0347719 (PMC13423031; doi:10.1371/journal.pone.0347719)

Supplementary File 4: Trace, Autocorrelation, and Posterior Predictive Check Plots for Model Evaluation


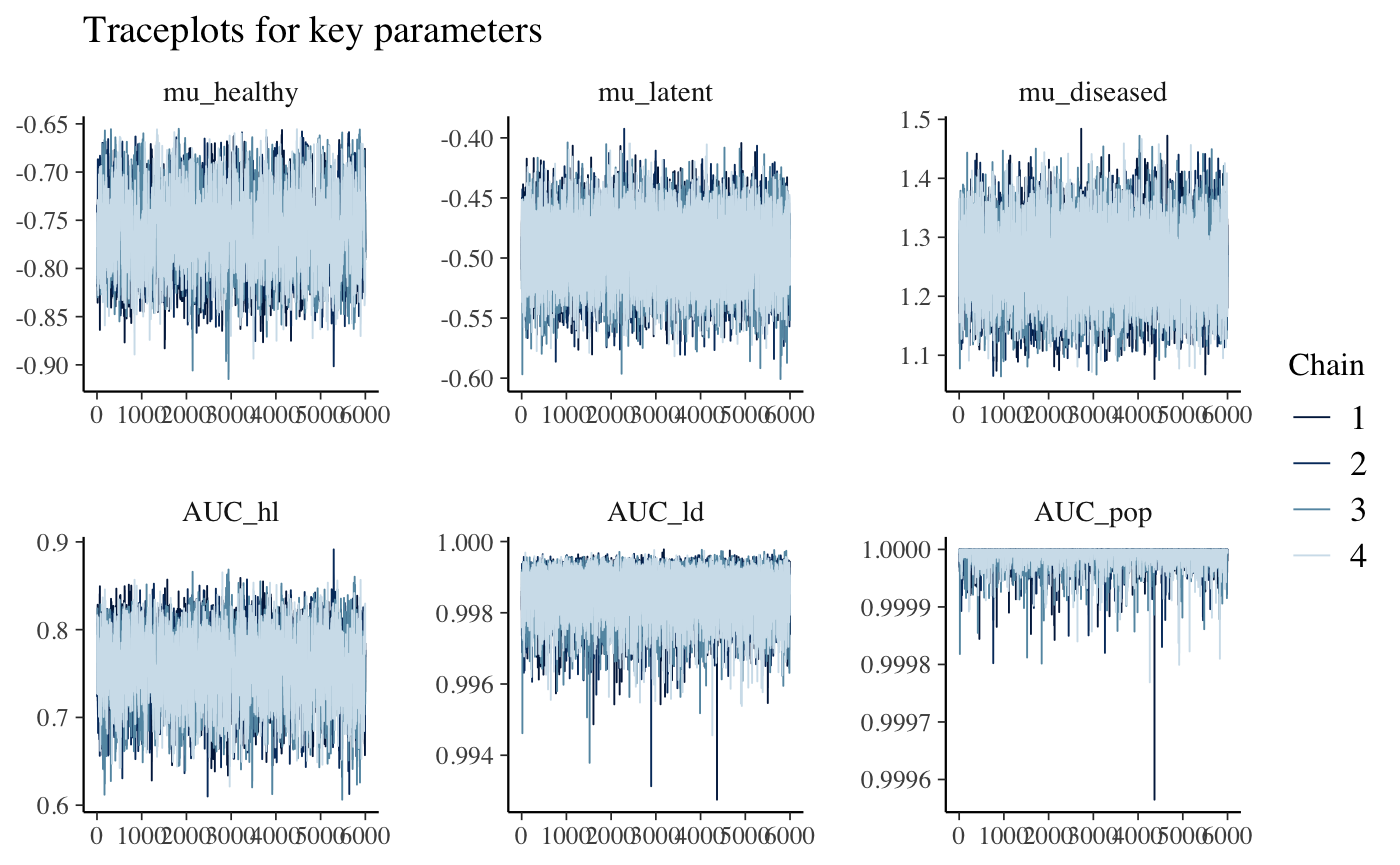


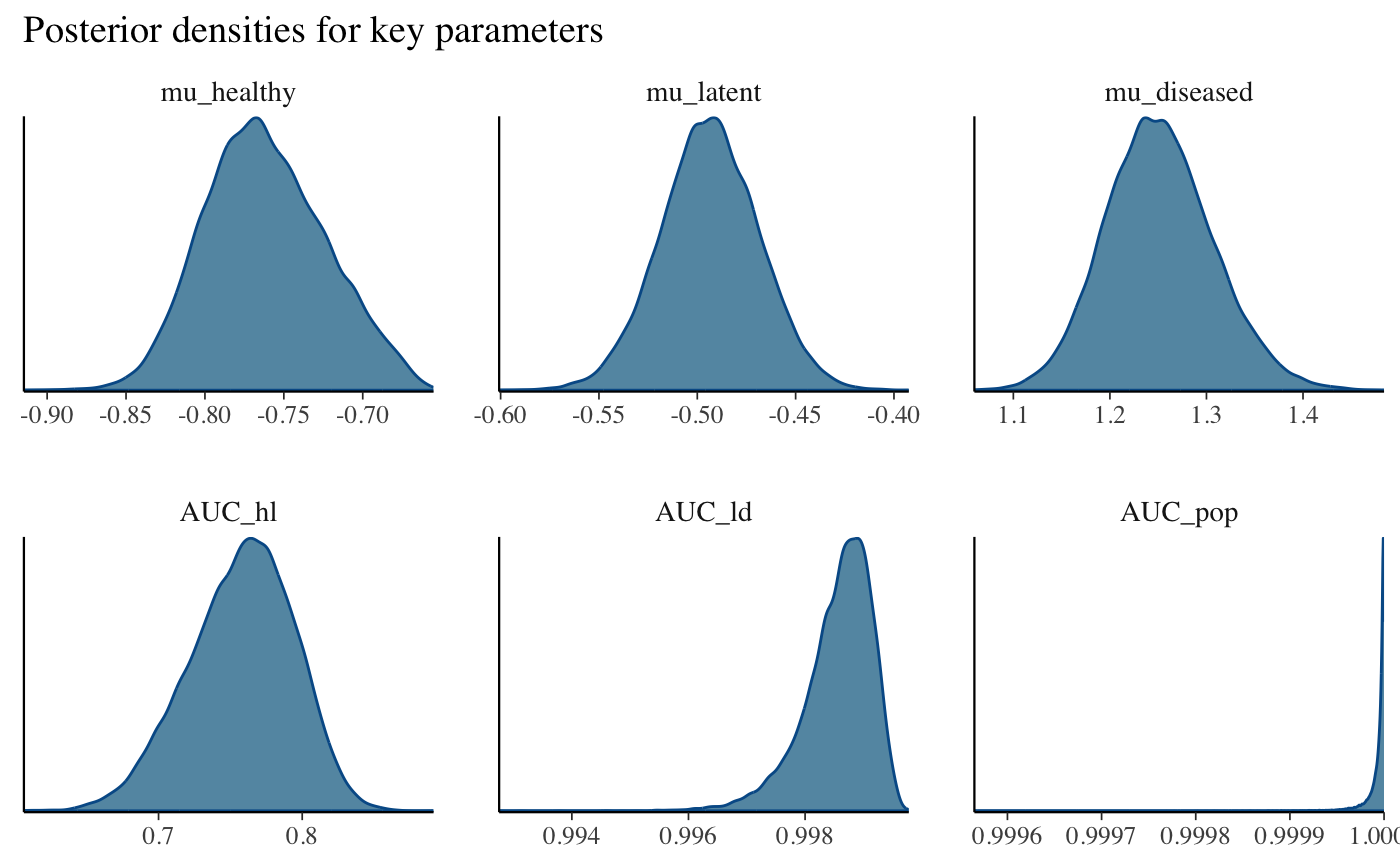


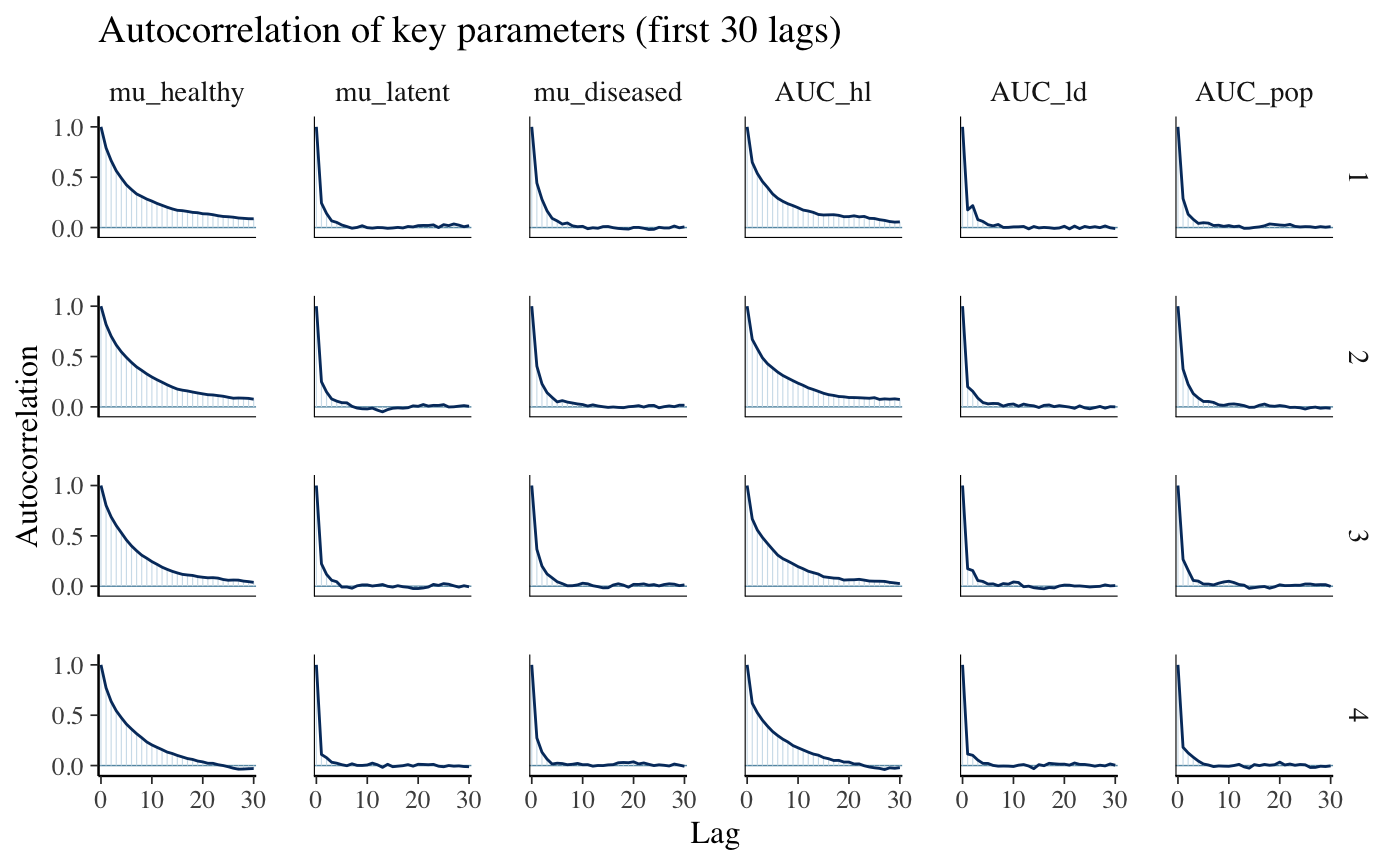


Posterior Predictive check


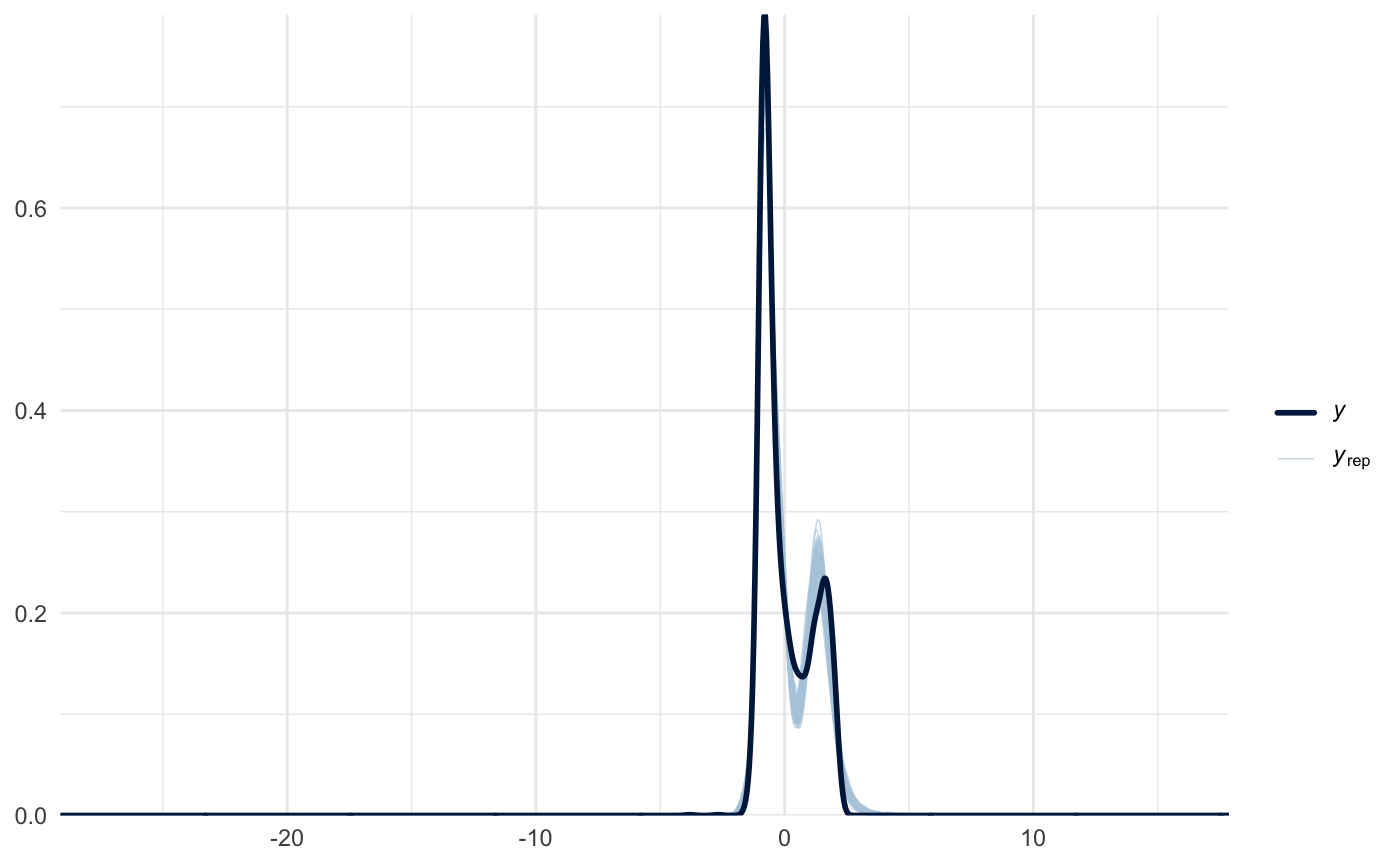

Supplement: S4 File — (DOC) [file pone.0347719.s004.doc]
