# Supplementary material for: Bayesian hierarchical mixture modelling to derive probabilistic iELISA thresholds for bovine brucellosis in endemic dairy systems
Source: PLoS One. 2026 Jul 30;21(7):e0347719. doi: 10.1371/journal.pone.0347719 (PMC13423031; doi:10.1371/journal.pone.0347719)
Supplement: Attachment — Submitted filename: PLOS One IMG_20260518_0001.pdf [file pone.0347719.s005.pdf]

Manuscript

Click here to access/download;Manuscript;Manuscript_PLOS.docx

Bayesian hierarchical mixture modelling to derive probabilistic iELISA thresholds for bovine brucellosis in endemic dairy systems

Authors:

Md. Shaffiul Alam[1], Md. Nazmul Islam[1], Bishwo Jyoti Adhikari[1], Shanta Islam[1], R S Mahmud[1], Md. Siddiqur Rahman[2], M. Ariful Islam[3], Muhammad Aktaruzzaman[3], Lefteris Meletis[4], Polychronis Kostoulas[4], A K M Anisur Rahman[1], *

Affiliations:

[1]Laboratory of Epidemiology & Preventive Medicine, Department of Medicine, Faculty of Veterinary Science, Bangladesh Agricultural University, Mymensingh-2202, Bangladesh

[2]Department of Medicine, Faculty of Veterinary Sciences, Bangladesh Agricultural University, Mymensingh-2202, Bangladesh

[3]Animal Welfare and Behaviour Laboratory, Department of Medicine, Bangladesh Agricultural University, Mymensingh-2202, Bangladesh

[4]Laboratory of Epidemiology & Artificial Intelligence, Faculty of Public Health, University of Thessaly, Volos, Greece

***Correspondence:** arahman_med@bau.edu.bd

Abstract

Background: In endemic dairy systems, the interpretation of serological tests for bovine brucellosis is compromised using fixed diagnostic cut-offs, which fail to account for continuous antibody distributions and population heterogeneity. This study aimed to apply a Bayesian hierarchical Gaussian mixture model (BHGMM) to resolve diagnostic uncertainty by deriving probabilistic, biologically informed thresholds for indirect ELISA (iELISA).

Methods: A cross-sectional dataset comprising 2,696 milk samples from large-scale dairy herds was analysed. Log-transformed and standardised antibody values were modelled using a three-component hierarchical mixture representing healthy, latent, and diseased populations. Posterior class distributions, herd-specific cut-offs, and prevalence were estimated, and model performance was evaluated using convergence diagnostics, posterior predictive checks, and ROC analysis.

Results: Three distinct serological populations were identified. Mean antibody levels (S/P%) were 5.29 in healthy, 17.07 in latent, and 299.84 in diseased animals. Dual diagnostic thresholds were estimated at 10.7 S/P% and 82.2 S/P%. Estimated class proportions were 23.5% healthy, 43.6% latent, and 32.9% diseased. Substantial between-herd heterogeneity was observed, with confirmatory cut-offs ranging from approximately 68 to 133 S/P% and herd-level true prevalence varying from about 1% to 67%. The model demonstrated high diagnostic accuracy (AUC = 84.5%) and stability across prior specifications.

Conclusions: Bayesian modelling captures intermediate serological "gray zones" and herd-level variability overlooked by standard binary interpretations. This probabilistic approach supports targeted control strategies in complex endemic environments.

Keywords: Healthy, Latent, Diseased, Gray zone, Dual cut-off, Herd-level heterogeneity

Introduction

Bovine brucellosis, caused primarily by *Brucella abortus*, remains a major zoonosis and a leading cause of reproductive failure in cattle worldwide. In endemic regions, the disease imposes a substantial economic burden on the dairy industry through abortion, infertility, and reduced milk yield, while simultaneously posing a serious public health threat to farmers, veterinary personnel, and consumers of unpasteurized dairy products [1–5]. The intensification of dairy sectors in these regions—characterized by high stocking densities and increased animal movement—creates favorable environments for the persistence and rapid transmission of *Brucella* spp [6]. Despite this, control programs in many endemic countries often lack strategic policies such as mass vaccination or systematic test-and-cull programs, leaving the growing commercial sector vulnerable.

Accurate diagnosis is central to effective control, particularly in intensive systems. The indirect enzyme-linked immunosorbent assay (iELISA) is commonly used for large-scale screening due to its high throughput and objectivity. However, standard interpretation typically applies a single manufacturer-recommended cut-off (e.g., S/P% 50%) to dichotomize results into positive or negative. This binary classification neglects the continuous and biologically complex nature of antibody responses. In endemic settings, many animals occupy a "gray zone" of intermediate antibody levels—representing latent or subclinical infections—that may fall below fixed positivity thresholds yet still contribute to transmission. Conversely, rigid cut-offs can lead to the unnecessary culling of productive animals due to false positives. While standard ELISA protocols often include a 'doubtful' category to reflect uncertainty near the cut-off, traditional epidemiological analyses frequently collapse this data into binary outcomes, discarding valuable nuance [7].

To address these limitations, statistical methods that treat serological outcomes as continuous distributions offer a superior fit to biological reality. Finite mixture models allow continuous serological measurements to be decomposed into latent biological subpopulations without requiring a perfect reference test. Embedding these models within a Bayesian hierarchical framework further enables estimation of herd-specific parameters while accounting for between-herd variability [8,9]. This approach is particularly relevant for large commercial herds in endemic areas, where infection pressure and background immunity differ substantially from settings where standard cut-offs were originally validated [10,11].

This study applied a Bayesian hierarchical Gaussian mixture modelling framework to: (1) identify serological subpopulations corresponding to healthy, latent, and diseased states; (2) derive herd-specific diagnostic thresholds; and (3) estimate true prevalence at both the animal and herd levels under diagnostic uncertainty.

Materials and Methods

Study design, target and study population

A cross-sectional study was conducted between January 2023 and December 2024. The target population consisted of dairy cattle within large-scale farming systems. The study population included a selection of institutional and private commercial dairy herds.

Sample size calculation and sampling protocol

Sample size calculations were performed using a one-stage cluster sampling approach via the 'epi.ssclus1estb' function in the epiR package [12] for R statistical environment (v4.5.1) [13]. The calculations were based on an expected true prevalence (P_y) of 20.4% [14] and a desired absolute precision ($\in$) of 5% with 95% confidence. To account for the clustering effect of animals within

herds, an intra-cluster correlation coefficient (ρ) of 0.09 [15] was applied. Assuming 100 animals per herd, the analysis initially indicated that 25 herds (totaling 2,473 animals) would be required to achieve the desired precision. In practice, however, the participating commercial herds were larger than expected. As a result, the target sample size at the animal level was not only met but exceeded, with 2,696 milk samples collected from 17 herds. This higher density of observations per herd provides robust data for characterizing the continuous serological distributions and ensures that the study remains adequately powered for the hierarchical mixture model, despite having fewer herds than originally planned [10].

Sample Collection and iELISA Testing

A total of 2,696 individual milk samples were collected from lactating cows across all participating herds. Samples were aseptically collected and stored at –20 °C until analysis. Antibodies against *Brucella* spp. were quantified using a commercially available antibody indirect ELISA (iELISA) assay (ID Screen® Brucellosis Indirect ELISA kit, Innovative Diagnostics, Grabels, France). Milk samples were centrifuged to separate lactoserum. The assay was performed according to manufacturer instructions. The results were interpreted by calculating the S/P% for each sample; samples with an S/P% less than or equal to 45% were classified as negative, those between 45% and 50% as doubtful, and those greater than 50% as positive [16].

Data Processing and Transformation

To address the positive skewness typical of serological data and to facilitate model convergence, the raw S/P ratio (SP%) values were log-transformed. A small constant c was added prior to transformation to handle zero or negative values, defined as $c = |min(SP)| + 1$

The transformed variable was defined as: $y_{ij} = log(SP_{ij} + c)$

where SP_{ij} represents the serological value for animal i in herd j.

What is this?

To place observations on a common scale and improve MCMC mixing, these log-transformed values were standardized to have a mean of 0 and a standard deviation of 1:

$$z_{ij} = \frac{y_{ij} - \bar{y}}{s_y}$$

Where $\bar{y}$ and s_y denote the sample mean and standard deviation of the log-transformed SP% values, respectively. All subsequent Bayesian analyses were conducted on the standardized variable z_{ij}.

Exploratory Component Selection

Prior to fitting the Bayesian model, the number of underlying biological populations was assessed using an unsupervised Gaussian Mixture Model (GMM) via the Expectation-Maximization algorithm (mclust package in R). Models ranging from 1 to 9 components were compared, with the optimal number selected based on the Bayesian Information Criterion (BIC). This analysis provided empirical evidence for a three-population structure:

- Healthy (low titers)
- Latent (intermediate titers)
- Diseased (high titers)

Similar pattern should be used for abbreviation for BHGMM everywhere in the manuscript

Hierarchical Bayesian Gaussian Mixture Model (HB-GMM)

Likelihood Specification

The likelihood of the observed standardized value z_{ij} or animal i in herd j is defined as a mixture of three density components [7]:

$$p(z_{ij} \mid \Theta) = \sum_{k=1}^{3} \pi_{jk} \cdot f_k(z_{ij} \mid \mu_{jk}, \sigma_k)$$

Where:

- π_{jk} is the herd-specific prevalence (mixing proportion) of component k in herd j.
- $f_k(\cdot)$ represents the probability density function for population$k$ k, defined as follows:

1. Healthy ($k = 1$) and Latent ($k = 2$) populations: Modeled as Normal distributions:

$$f_k(z_{ij} \mid \mu_{jk}, \sigma_k) = \frac{1}{\sigma_k \sqrt{2\pi}} \exp\left(-\frac{(z_{ij} - \mu_{jk})^2}{2\sigma_k^2}\right)$$

2. Diseased Population $k = 3$: To account for heavy tails and high-titer outliers, the density for the diseased population is modeled using a Student-t distribution with 4 degrees of freedom [10]:

$$f_3(z_{ij} \mid \mu_{j3}, \sigma_3) = \frac{\Gamma\left(\frac{\nu+1}{2}\right)}{\Gamma\left(\frac{\nu}{2}\right)\sqrt{\nu\pi}\sigma_3}\left[1 + \frac{(z_{ij} - \mu_{j3})^2}{\nu\sigma_3^2}\right]^{-\frac{\nu+1}{2}}, \quad \nu = 4$$

Ordered Population Means and Non-centered Parameterization

To enforce biological consistency ($Healthy < Latent < Diseased$), we applied an ordering constraint using positive offset parameters (δ):

$$\mu_2 = \mu_1 + \delta_1$$

$$\mu_3 = \mu_2 + \delta_2$$

To account for the hierarchical clustering of animals within herds and to improve the efficiency of Markov Chain Monte Carlo (MCMC) sampling, we applied a non-centered parameterization for the herd-specific means. This is mathematically expressed as: $\mu_{(jk)}$:

$$\mu_{(jk)} = \mu_k + \sigma_{(\mu_k)} \cdot \eta_{(jk)}, \quad \eta_{(jk)} \sim N(0,1)$$

Where: μ_{jk} is the estimated mean antibody level for component k in herd j. μ_k represents the global, population-level mean for component k. σ_{μ_k} is the between-herd standard deviation, quantifying the expected heterogeneity in mean antibody levels across herds for component k. η_{jk} represents the latent herd-effect offset for herd j, drawn from a standard normal distribution with a mean of 0 and a variance of 1.

Herd-Specific Parameters and Cutoff Determination

Herd-Specific Prevalence Estimation

The distribution of animals across the three classes within each herd was modeled using a Dirichlet prior:

$$\pi_j \sim \text{Dirichlet}(\alpha_1, \alpha_2, \alpha_3)$$

This allows the model to estimate herd-specific prevalence while sharing information across the entire population (shrinkage estimation).

Localized Cutoff Logic

Rather than a single global cutoff, our model allows for herd-specific thresholds. The cutoff between class k and class $k+1$ for herd j is the point C where the weighted densities intersect [8]:

$$f_k\left(C_{(j,k|k+1)}\right) \cdot \pi_{(jk)} = f_{(k+1)}\left(C_{(j,k|k+1)}\right) \cdot \pi_{(j,k+1)}$$

This ensures that the diagnostic threshold adapts to the specific disease pressure and titer distribution of the local environment.

Model Implementation and Convergence Diagnostics

We implemented the model in Stan [17] using the 'cmdstanr' interface [18] within the R statistical environment (v4.5.1) [13]. Posterior samples were drawn using the No-U-Turn Sampler (NUTS) across four parallel chains, each consisting of 2,000 warmup and 6,000 sampling iterations (totaling 24,000 post-warmup samples). Convergence was assessed through visual inspection of trace plots to confirm stationarity and mixing. Additionally, we monitored Effective Sample Size (ESS) to ensure sufficient posterior exploration, requiring both Bulk-ESS and Tail-ESS to exceed 400. All parameters met current best practices for MCMC convergence, with a potential scale reduction factor R-hat $(\widehat{R})$ maintained below 1.01 [19].

Performance Evaluation

Posterior Predictive Checks (PPC)

The model's generative fit was validated by simulating replicated datasets (y_{rep}) from the posterior predictive distribution [10]:

$$z_{(ij)}^{(rep)} \sim p\left(z^{(rep)} \middle| y\right)$$

The observed Log-SP% distribution was compared against the 95% credible intervals of the replicated data to ensure the model accurately captured the data structure.

Diagnostic Accuracy (AUC)

Diagnostic accuracy was evaluated empirically to quantify the separability of the three identified subpopulations. Pairwise Area Under the Curve (AUC) values were calculated for three distinct comparisons: Healthy ($k = 1$) versus Subclinical ($k = 2$), Subclinical ($k = 2$) versus Diseased ($k = 3$), and Healthy ($k = 1$) versus Diseased ($k = 3$). Because the diseased component was modeled using a heavy-tailed Student-t distribution, closed-form analytical calculation of the AUC is intractable; therefore, accuracy was estimated computationally. Using the posterior predictive distributions, random draws from the estimated densities of each class pair were compared across all possible classification thresholds to compute the empirical AUC. This pairwise evaluation was implemented via the pROC package in R [20].

Prior Specification and Sensitivity Analysis

Data Standardization and Parameterization

Standardization is standard practice in hierarchical mixture modeling to improve the geometry of the posterior distribution, thereby increasing the efficiency of the Markov chain Monte Carlo (MCMC) sampling [10]. To resolve the "label switching" non-identifiability problem inherent in finite mixture models, we imposed an ordering constraint on the component means ($Healthy < Latent < Diseased$) by defining the means of the Latent and Diseased classes as positive offsets (δ) from the preceding class.

Prior selection

The manufacturer's interpretation for iELISA defines distinct zones (Negative 45%, Doubtful 45–50%, Positive > 50%), implying a biologically plausible separation between the antibody distributions of healthy and infected populations [21]. Consistent with this biological knowledge

and statistical guidance for regularization, the separation parameter (δ) was assigned a weakly informative Normal prior centered on moderate class separation$\mu = 0.6, \sigma = 0.3$ Variance parameters were assigned bounded Half-Normal hyperpriors, and class prevalences were modeled using a Dirichlet distribution.

Sensitivity Analysis

To ensure that our results were driven by the data rather than subjective prior choices, we performed a global sensitivity analysis comparing three distinct prior scenarios [22]. The Primary Model utilized the priors described above, including a Dirichlet concentration parameter of $\alpha = 2$ to favor non-zero prevalence across all classes. This was contrasted with a Weakly Informative Scenario, which employed broad, flat priors (e.g., $\sigma = 5.0$ for means) and a uniform Dirichlet prior ($\alpha = 1$) to minimize the influence of regularization. Finally, we evaluated a Strong Informative Scenario characterized by highly concentrated priors (small σ) for the separation parameter (δ) to test model stability under restrictive assumptions. We monitored the stability of the posterior distributions for prevalence and the Area Under the Curve (AUC) across these scenarios. Consistency in estimates across these diverse prior specifications was interpreted as evidence that the likelihood (the data) dominated the statistical inference. All data, model, code and diagnostic plots are provided in Supplementary Materials S1–S4.

Use of Artificial Intelligence

We utilized ChatGPT (OpenAI, GPT-5.2, web version) to assist with English language editing and enhance clarity of expression. All scientific content, analyses, and interpretations are solely the responsibility of the authors.

Results

Descriptive statistics

The age of the sampled cows ranged from 2.4 to 17.5 years, with a mean age of 6.33 years and a median of 6.0 years. The interquartile range (IQR) spanned from 4.0 to 8.0 years. The breed composition of the study population (N=2,696) was predominantly Local x Friesian, comprising 2,436 animals (90.4%). The remaining herds consisted of Local x Sahiwal (n=150; 5.6%) and Local x Jersey (n=110; 4.1%).

Exploratory Analysis and Model Convergence

Unsupervised Gaussian mixture modelling using the Bayesian Information Criterion supported a three-component solution. The components corresponded to distributions consistent with Healthy, Latent, and Diseased subpopulations. The hierarchical Bayesian model achieved convergence across all monitored parameters, with potential scale reduction factors $\hat{R} \leq 1.01$ and effective sample sizes exceeding 1000.

Diagnostic Performance and Model Validation

The estimated population-level area under the receiver operating characteristic curve was 84.5% (95% credible interval: 79.9–88.6) (Table 1; Figure 1). Posterior predictive checks showed that the observed log-transformed S/P% distribution lay within the 95% credible intervals of replicated datasets generated from the posterior distribution (Figure 2).

Table 1. Posterior estimates of diagnostic performance metrics for the iELISA assay based on the derived dual-cutoff system.

Metric	Definition	Estimate (95% CrI)
**Sp_1 (Healthy)**	Specificity at the lower cut-off (C1); probability that a truly healthy animal tests below C1.	98.12% (86.30–100.00)

**Se_1 (Screening)**	Sensitivity at C1; probability that an infected animal (latent or diseased) tests above C1.	80.50% (74.20–86.60)
**Se_2 (Diseased)**	Sensitivity at the upper cut-off (C2); probability that a diseased animal tests above C2.	95.10% (92.70–97.10)
**Sp_2 (Latent)**	Specificity at C2; probability that latent animals test below C2 and are not classified as diseased.	98.10% (96.60–99.10)
**Latent detection (Gray zone)**	Proportion of latent animals with results between C1 and C2.	62.00% (58.00–65.50)
**False positive classification**	Probability that a healthy animal is incorrectly classified as diseased (result $\geq$ C2).	0.00% (0.00–0.00)
**False negative classification**	Probability that a diseased animal is incorrectly classified as healthy (result < C1).	0.70% (0.40–1.00)

A dual-cutoff classification framework was estimated. At the lower cutoff (C1), specificity for identifying healthy animals was 98.12% (95% CrI: 86.30–100.00). At the upper cutoff (C2), sensitivity for identifying diseased animals was 95.10% (95% CrI: 92.70–97.10) (Table 1). Within the intermediate range (C1 < test value < C2), 62.00% of latent animals were classified in the gray zone (95% CrI: 58.00–65.50). Posterior estimates of AUC and component means were similar across informative, weakly informative, and vague prior specifications (Table 2).

Table 2. Results of a sensitivity analysis on the prior information utilized to determine the cutoff values for the indirect ELISA in classifying healthy, latent, and diseased subpopulations

Scenario	Parameter	Posterior mean	SD	Median	95% CrI
Main analysis	μ Healthy	−0.77	0.04	−0.77	−0.84 to −0.69
	μ Latent	−0.51	0.02	−0.51	−0.56 to −0.47
	μ Diseased	1.25	0.05	1.23	1.13 to 1.34
Weakly informative priors	μ Healthy	−0.76	0.04	−0.76	−0.83 to −0.69
	μ Latent	−0.51	0.02	−0.507	−0.554 to −0.458
	μ Diseased	1.25	0.06	1.25	1.14 to 1.37
	AUC	0.999	0.00001	0.999	0.999 to 1.00
Strong separation priors	μ Healthy	−0.78	0.04	−0.778	−0.838 to −0.697
	μ Latent	−0.52	0.02	−0.521	−0.566 to −0.479

μ Diseased	1.19	0.05	1.198	1.104 to 1.301

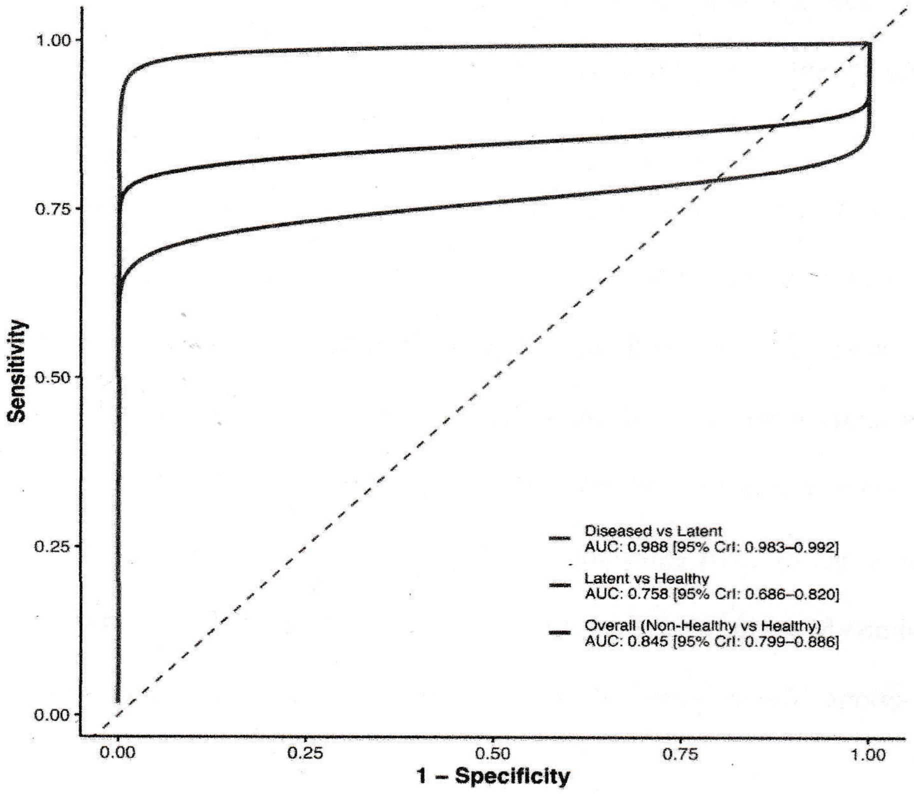

Figure 1. Receiver Operating Characteristic (ROC) curve illustrating the classification performance of the Bayesian Hierarchical Gaussian Mixture Model (HGMM). The curve demonstrates the trade-off between sensitivity and specificity for distinguishing healthy, latent, and diseased animals, with the area under the curve (AUC) quantifying overall model accuracy

Population-Level Parameter Estimates

Posterior component means on the original S/P% scale differed across the three classes (Table 3). The mean S/P% was 5.21 (95% CrI: 2.71–8.31) for the Healthy class, 17.04 (95% CrI: 14.63–19.56) for the Latent class, and 299.47 (95% CrI: 262.81–344.08) for the Diseased class. Estimated weighted class prevalences were 23.47% (95% CrI: 21.7–25.3) for Healthy, 43.61% (95% CrI: 41.2–46.0) for Latent, and 32.91% (95% CrI: 30.7–35.1) for Diseased animals.

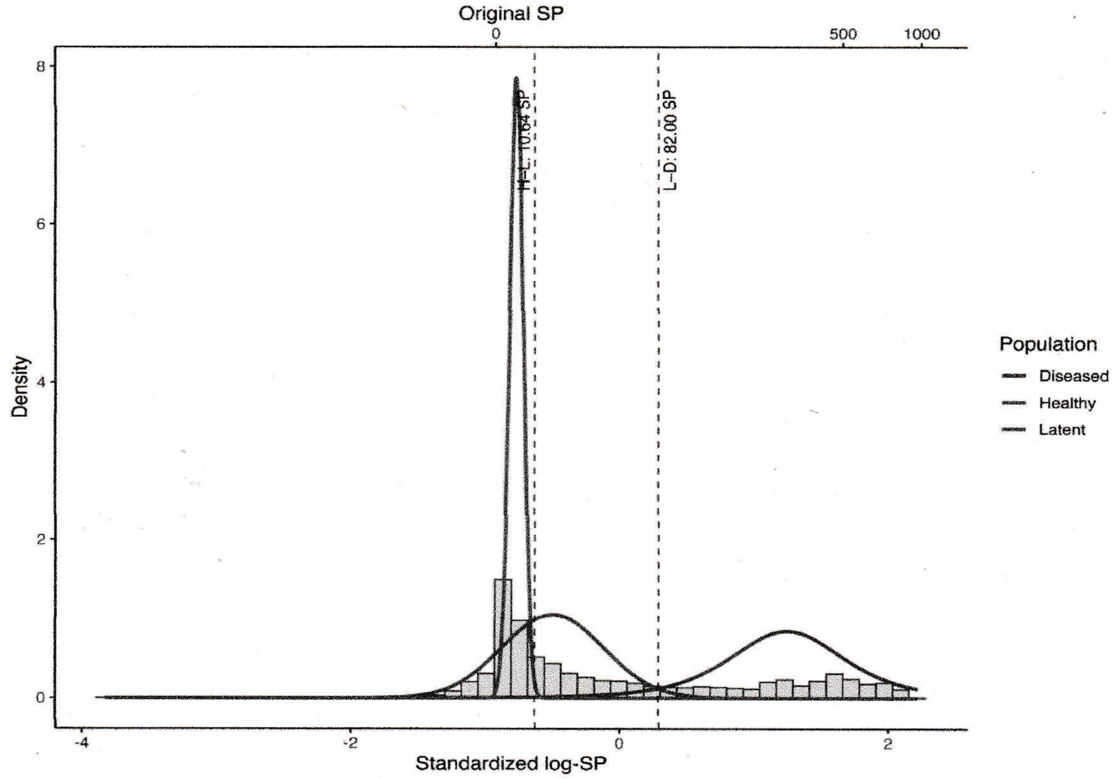

Figure 2. Posterior predictive distributions of S/P% values for the Healthy, Latent, and Diseased classes estimated using the Bayesian hierarchical Gaussian mixture model. The estimated lower cutoff separating Healthy from Latent animals was 10.64 S/P% (95% CrI: 8.82–12.71), and the upper cutoff separating Latent from Diseased animals was 82.00 S/P% (95% CrI: 67.76–102.16).

Table 3. Posterior estimates of the component means (μ) in the standardized log scale and the corresponding original S/P scale for the three-component Bayesian Hierarchical Gaussian Mixture Model

Class	Standardized mean (95% Credible Interval)	S/P mean (95% Credible Interval)
Healthy	-0.76 (-0.83–-0.68)	5.29 (2.78–8.34)
Latent	-0.49 (-0.54–-0.44)	17.07 (14.62–19.65)
Diseased	1.25 (1.15–1.37)	299.84 (263.01–345.75)

Table 4. Posterior mean estimates and 95% credible intervals for the population-level prevalence of Healthy, Latent, and Diseased classes, weighted by herd size.

Class	Mean (95% Credible Interval)
**Healthy**	23.48% (21.70–25.30)
**Latent**	43.61% (41.20–46.00)
**Diseased**	32.91% (30.70–35.10)

Herd-Specific Analysis

Herd-level variation was observed in confirmatory cutoff estimates and true milk antibody prevalence.

Herd-specific upper cutoffs (C2) ranged from 67.62 in Herd 1 to 132.76 in Herd 3 (Figure 3; Table 5). Estimated true milk antibody prevalence also varied across herds, from 1.05% (95% CrI: 0.13–2.86) in Herd 14 to 67.12% (95% CrI: 62.49–71.62) in Herd 17 (Table 6).

Table 5. Herd specific cutoff values and their 95% Credible intervals

Herd	Mean Cutoff	95% Credible Interval
Herd 1 (n=42)	67.62	51.06 – 89.04
Herd 2 (n=13)	70.36	38.96 – 102.79
Herd 3 (n=136)	132.76	102.74 – 171.74
Herd 4 (n=51)	102.56	76.49 – 139.36
Herd 5 (n=485)	101.68	82.56 – 129.70
Herd 6 (n=247)	97.78	80.55 – 115.98
Herd 7 (n=76)	119.52	98.42 – 143.90
Herd 8 (n=53)	120.34	92.16 – 150.10
Herd 9 (n=179)	94.06	75.21 – 121.87
Herd 10 (n=36)	117.00	87.23 – 155.50
Herd 11 (n=102)	93.98	74.02 – 115.86
Herd 12 (n=57)	104.00	77.31 – 137.78
Herd 13 (n=148)	106.71	87.04 – 130.27
Herd 14 (n=184)	83.20	44.59 – 130.75
Herd 15 (n=39)	122.71	81.61 – 176.46
Herd 16 (n=38)	72.59	49.21 – 97.85
Herd 17 (n=810)	89.35	75.56 – 104.52

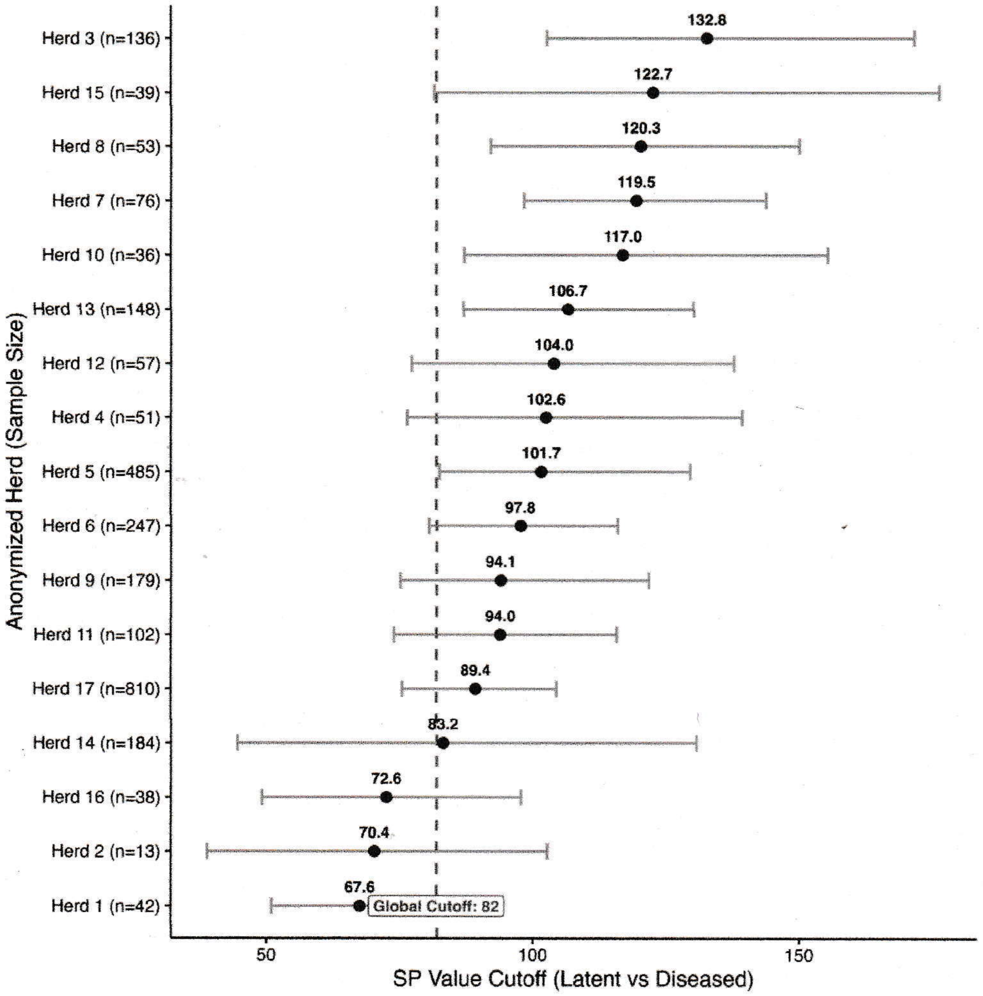

Figure 3. Herd-specific confirmatory cutoffs (C2) for the iELISA test estimated using the Bayesian hierarchical Gaussian mixture model. Points represent posterior mean cutoffs for each herd, and the horizontal dashed line indicates the overall population-level cutoff (82.00 S/P%)

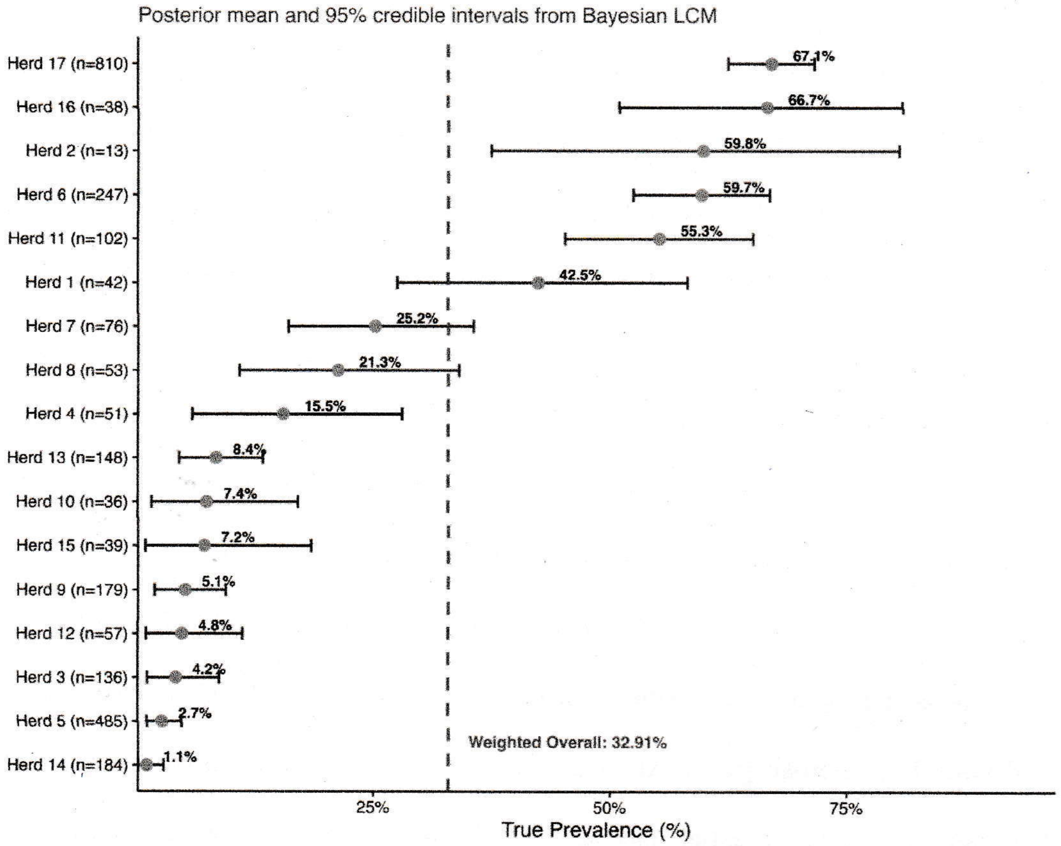

Figure 4. Herd-level prevalence of bovine brucellosis estimated using a Bayesian hierarchical Gaussian mixture model. Each point represents the posterior mean prevalence for a herd, with 95% credible intervals reflecting uncertainty in the estimates

Table 6. Posterior estimates of the within-herd true milk antibody prevalence of brucellosis derived from the Bayesian Hierarchical Gaussian Mixture Model

**Herd** (Tested)	**Mean True Prevalence (95% Credible Interval)**
Herd 1 (n=42)	42.46% (27.55–58.23%)
Herd 2 (n=13)	59.84% (37.47–80.56%)
Herd 3 (n=136)	4.16% (1.16–8.76%)
Herd 4 (n=51)	15.49% (5.86–28.05%)
Herd 5 (n=485)	2.69% (1.08–4.72%)
Herd 6 (n=247)	59.70% (52.47–66.89%)
Herd 7 (n=76)	25.20% (16.02–35.55%)
Herd 8 (n=53)	21.29% (10.87–34.04%)
Herd 9 (n=179)	5.13% (1.91–9.42%)
Herd 10 (n=36)	7.45% (1.62–17.07%)
Herd 11 (n=102)	55.26% (45.27–65.15%)
Herd 12 (n=57)	4.80% (1.02–11.19%)
Herd 13 (n=148)	8.42% (4.49–13.36%)

Herd 14 (n=184)	1.05% (0.13–2.86%)
Herd 15 (n=39)	7.16% (0.91–18.42%)
Herd 16 (n=38)	66.66% (51.02–80.95%)
Herd 17 (n=810)	67.12% (62.49–71.62%)
**Weighted Overall**	**32.91% (30.73–35.06%)**

Discussion

This study demonstrates the utility of Bayesian hierarchical mixture modelling for characterizing heterogeneous serological responses to bovine brucellosis in intensive dairy herds. By deriving probabilistic diagnostic thresholds, this approach overcomes the limitations of conventional fixed cut-offs, which often fail in endemic settings.

A key finding was the identification of a substantial "latent" subpopulation, comprising approximately 44% of sampled animals. These individuals exhibited antibody titres elevated above the healthy baseline yet lower than the diseased cluster. This pattern aligns with the intracellular pathogenesis of *Brucella abortus*, where persistence within macrophages drives fluctuating humoral responses and prolonged subclinical infection phases [23,24]. In traditional binary testing, these intermediate animals are forced into a dichotomous classification, resulting in "false negatives" that sustain transmission or "false positives" that cause economic waste [25].

The high proportion of animals in non-negative categories (Latent + Diseased) suggests a state of "endemic stability" [26]. In high-transmission environments, constant re-exposure likely acts as a natural booster, maintaining high antibody prevalence with muted clinical signs. However, the identification of this latent class challenges the assumption that low or borderline titers represent merely "background noise." Animals in this “grey zone” may harbor persistent infections, acting as cryptic reservoirs that shed bacteria and could contribute to public health risks [27,28]. The BHGMM’s dual-cutoff framework is therefore an epidemiological necessity, identifying silent carriers that standard binary approaches overlook [29].

Methodologically, the model demonstrated high diagnostic discrimination. The use of a Student-t distribution for the diseased component was critical to accommodate biological outliers without distorting estimates [8,10,11,30]. Furthermore, sensitivity analyses confirmed that classification was driven by observed data rather than prior specifications, satisfying key criteria for robust Bayesian inference [31].

A major strength of the hierarchical framework was its ability to quantify herd-level heterogeneity. Confirmatory cut-offs varied substantially between herds, proving that universal thresholds generate systematic misclassification in endemic settings [21]. This variability supports the move toward risk-based surveillance strategies tailored to specific herd epidemiology [32].

These findings have direct implications for brucellosis control globally. First, binary serological interpretation weakens control programs by allowing subclinically infected animals to evade detection [23]. Second, the heterogeneity in herd-specific thresholds supports international recommendations to adapt decision thresholds to the local context [33]. Finally, the large latent population suggests that testing alone is insufficient; effective control requires integrated programs combining vaccination, movement control, and strategic testing [34,35]. From a One Health perspective, detecting subclinical infection is critical to reducing zoonotic transmission [36]. Incorporating Bayesian modelling into routine surveillance is a practical step toward aligning analytics with the complex ecological reality of zoonotic disease [37].

Several limitations should be noted. Serological results were analyzed without concurrent bacteriological confirmation. The cross-sectional design precludes tracking temporal progression between states. While the study focused on intensive herds, the methodological framework is applicable to other production systems.

Conclusion

Bayesian hierarchical mixture modelling of iELISA data revealed substantial serological heterogeneity and a large intermediate antibody population that is obscured by fixed cut-offs. The identification of herd-specific diagnostic thresholds provides a robust framework for improving surveillance accuracy. Probabilistic, herd-adapted diagnostic frameworks are recommended to support targeted brucellosis control within integrated One-Health programs in endemic regions.

Declarations

Ethics approval

This study involved sampling of dairy cattle and did not include human participants. All procedures were conducted in accordance with internationally accepted standards for the ethical use of animals in research and reporting guidelines for diagnostic accuracy studies using Bayesian latent class models (STARD-BLCM). Ethical approval was obtained from the Animal Welfare and Experimentation Ethical Committee of Bangladesh Agricultural University (approval no. AWEEC/BAU/2022/07).

Consent to participate

Not applicable

Consent for publication

Not applicable.

Availability of data and materials

The datasets, model code, and analysis scripts supporting the conclusions of this article are available as Supplementary Material.

Competing interests

The authors declare no conflict of interest.

Funding

This research was funded by the Livestock and Dairy Development Project (LDDP), grant number 2022/7/LDDP. The funder had no role in study design, data collection, analysis, interpretation of the results, manuscript preparation, or the decision to submit the work for publication.

Authors' contributions

A.K.M. Anisur Rahman (AKMAR), Md. Siddiqur Rahman (MSR), M. Ariful Islam (MAI), and Polychronis Kostoulas (PK) conceived and supervised the study. Md. Shaffiul Alam (MSA), Bishwo Jyoti Adhikari (BJA), Shanta Islam (SI), R.S. Mahmud (RSM), Muhammad Aktaruzzaman (MA), and Md. Nazmul Islam (MNI) contributed to study design, data curation, and formal analysis. AKMAR, MSA, Lefteris Meletis (LM), and PK developed and implemented the Bayesian hierarchical mixture model. BJA, SI, MNI, MA, and RSM conducted field investigation, sample collection, and laboratory testing. AKMAR and MSR acquired funding and provided resources. MSA, MA, MNI, SI, BJA, and RSM drafted the original manuscript. AKMAR, MAI, MSR, LM, and PK critically revised the manuscript.

Acknowledgements

We are grateful to the farmers for dedicating their time and providing samples from their animals.

References

1. Rasmussen P, Barkema HW, Osei PP, Taylor J, Shaw AP, Conrady B, et al. Global losses due to dairy cattle diseases: A comorbidity-adjusted economic analysis. Journal of Dairy Science. 2024;107: 6945–6970.

2. Pal M, Gizaw F, Fekadu G, Alemayehu G, Kandi V. Public Health and Economic Importance of Bovine Brucellosis: An Overview. AJEID. 2017;5: 27–34. doi:10.12691/ajeid-5-2-2

3. Mitiku W, Desa G. Review of Bovine Brucellosis and Its Public Health Significance. HR. 2020;1: 16–33. doi:10.47285/hr.v1i2.62

4. Khurana SK, Sehrawat A, Tiwari R, Prasad M, Gulati B, Shabbir MZ, et al. Bovine brucellosis – a comprehensive review. Veterinary Quarterly. 2021;41: 61–88. doi:10.1080/01652176.2020.1868616

5. Santos RL, Martins TM, Borges ÁM, Paixão TA. Economic losses due to bovine brucellosis in Brazil. Pesq Vet Bras. 2013;33: 759–764. doi:10.1590/S0100-736X2013000600012

6. Samad MA. A SIX-DECADE REVIEW: RESEARCH ON CATTLE PRODUCTION, MANAGEMENT AND DAIRY PRODUCTS IN BANGLADESH. JVMOHR. 2020;2. doi:10.36111//jvmohr.2020.2(2).0021

7. Yang DA, Xiao X, Jiang P, Pfeiffer DU, Laven RA. Keeping continuous diagnostic data continuous: Application of Bayesian latent class models in veterinary research. Preventive veterinary medicine. 2022;201: 105596.

8. McLachlan GJ, Lee SX, Rathnayake SI. Finite Mixture Models. Annu Rev Stat Appl. 2019;6: 355–378. doi:10.1146/annurev-statistics-031017-100325

9. Mathevon Y, Foucras G, Falguières R, Corbiere F. Estimation of the sensitivity and specificity of two serum ELISAs and one fecal qPCR for diagnosis of paratuberculosis in sub-clinically infected young-adult French sheep using latent class Bayesian modeling. BMC Vet Res. 2017;13: 230. doi:10.1186/s12917-017-1145-x

10. Andrew Gelman, John B. Carlin, Hal S. Stern, David B. Dunson, Aki Vehtari, And Donald B. Rubin. Bayesian data analysis. Third edition. CRC Press; 2013.

11. Branscum A, Gardner I, Johnson W. Bayesian modeling of animal-and herd-level prevalences. Preventive veterinary medicine. 2004;66: 101–112.

12. Stevenson M, Nunes T, Heuer C, Marshall J, Sanchez J, Thornton R, et al. epiR: tools for the analysis of epidemiological data. R package version. 2018;2: 26.

13. R Core Team. R: A language and environment for statistical computing. R Foundation for Statistical Computing, Vienna, Austria. http://www R-project org/. 2025.

14. Rahman AKMA, Smit S, Devleesschauwer B, Kostoulas P, Abatih E, Saegerman C, et al. Bayesian evaluation of three serological tests for the diagnosis of bovine brucellosis in Bangladesh. Epidemiol Infect. 2019;147: e73. doi:10.1017/S0950268818003503

15. Otte M, Gumm I. Intra-cluster. Preventive Veterinary Medicine. 1997;3: 147–150.

When interpreting iELISA results for testing brucellosis, why the reference is for Paratuberculosis?

16. Kibria AM, Islam MN, Alam MS, Adhikari BJ, Islam S, Rafeh AA, et al. Estimated true prevalence and associated risk factors of bovine paratuberculosis antibodies in dairy herds in Bangladesh. PLoS One. 2026;21: e0334683.

17. Carpenter B, Gelman A, Hoffman MD, Lee D, Goodrich B, Betancourt M, et al. Stan: A probabilistic programming language. Journal of statistical software. 2017;76: 1–32.

18. Gabry J. cmdstanr: R Interface to'CmdStan'. (No Title). 2021.

19. Vehtari A, Gelman A, Simpson D, Carpenter B, Bürkner P-C. Rank-normalization, folding, and localization: An improved Rˆ for assessing convergence of MCMC (with discussion). Bayesian analysis. 2021;16: 667–718.

20. Robin X, Turck N, Hainard A, Tiberti N, Lisacek F, Sanchez J-C, et al. Package 'pROC.' Package "pROC. 2021.

21. World Organisation for Animal Health. Principles and methods of validation of diagnostic assays for infectious diseases. Paris; 2024. Available: https://www.woah.org/en/what-we-do/standards/codes-and-manuals/terrestrial-manual-online-access/

22. Berkvens D, Speybroeck N, Praet N, Adel A, Lesaffre E. Estimating disease prevalence in a Bayesian framework using probabilistic constraints. Epidemiology. 2006;17: 145–153.

23. Godfroid J, Scholz H, Barbier T, Nicolas C, Wattiau P, Fretin D, et al. Brucellosis at the animal/ecosystem/human interface at the beginning of the 21st century. Preventive veterinary medicine. 2011;102: 118–131.

24. Nielsen K. Diagnosis of brucellosis by serology. Veterinary microbiology. 2002;90: 447–459.

25. Greiner M, Sohr D, Göbel P. A modified ROC analysis for the selection of cut-off values and the definition of intermediate results of serodiagnostic tests. Journal of immunological methods. 1995;185: 123–132.

26. Coleman P, Perry BD, Woolhouse ME. Endemic stability—a veterinary idea applied to human public health. The Lancet. 2001;357: 1284–1286.

27. Islam MdS, Islam MdA, Khatun MstM, Saha S, Basir MdS, Hasan M-M. Molecular Detection of *Brucella* spp. from Milk of Seronegative Cows from Some Selected Area in Bangladesh. Journal of Pathogens. 2018;2018: 1–7. doi:10.1155/2018/9378976

28. Gwida M, El-Ashker M, Melzer F, El-Diasty M, El-Beskawy M, Neubauer H. Use of serology and real time PCR to control an outbreak of bovine brucellosis at a dairy cattle farm in the Nile Delta region, Egypt. Irish veterinary journal. 2015;69: 3.

29. Ozsvari L, Harnos A, Lang Z, Monostori A, Strain S, Fodor I. The Impact of Paratuberculosis on Milk Production, Fertility, and Culling in Large Commercial Hungarian Dairy Herds. Front Vet Sci. 2020;7: 565324. doi:10.3389/fvets.2020.565324

Follow journal guidelines

30. Bonfini B, Chiarenza G, Paci V, Sacchini F, Salini R, Vesco G, et al. Cross-reactivity in serological tests for brucellosis: a comparison of immune response of Escherichia coli O157: H7 and Yersinia enterocolitica O: 9 vs Brucella spp. 2018.

31. Hamra G, MacLehose R, Richardson D. Markov chain Monte Carlo: an introduction for epidemiologists. International journal of epidemiology. 2013;42: 627–634.

32. Stärk KD, Regula G, Hernandez J, Knopf L, Fuchs K, Morris RS, et al. Concepts for risk-based surveillance in the field of veterinary medicine and veterinary public health: Review of current approaches.

33. Ghanbari MK, Gorji HA, Behzadifar M, Sanee N, Mehedi N, Bragazzi NL. One health approach to tackle brucellosis: a systematic review. Tropical medicine and health. 2020;48: 86.

34. Abd El-Wahab EW. A scoping review of the national strategy for brucellosis control in Egypt: logic framework, challenges, and prospects. One Health Outlook. 2025;7: 42.

35. Al Hamada A, Bruce M, Barnes A, Habib I, D. Robertson I. Cost–benefit analysis of a mass vaccination strategy to control brucellosis in sheep and goats in northern Iraq. Vaccines. 2021;9: 878.

36. McDermott J, Grace D, Zinsstag J. Economics of brucellosis impact and control in low-income countries. Revue scientifique et technique (International Office of Epizootics). 2013;32: 249–261.

37. Zhou K, Wu B, Pan H, Paudyal N, Jiang J, Zhang L, et al. ONE health approach to address zoonotic brucellosis: a spatiotemporal associations study between animals and humans. Frontiers in Veterinary Science. 2020;7: 521.

Supplementary Files Caption

Supplementary File 1. Raw dataset containing iELISA S/P values and herd ID used for the Bayesian Hierarchical Gaussian Mixture Model analysis (.csv format).

Supplementary File 2. Stan code for the Bayesian Hierarchical Gaussian Mixture Model (HB-GMM) including likelihood definitions, priors, and non-centered parameterizations (.doc format).

Supplementary File 3. R script for data preprocessing, model execution via 'cmdstanr', and posterior predictive checks (.doc format).

Supplementary File 4: Trace, Autocorrelation, and Posterior Predictive Check Plots for Model Evaluation (.doc format)
