# Supplementary material for: Bayesian hierarchical mixture modelling to derive probabilistic iELISA thresholds for bovine brucellosis in endemic dairy systems
Source: PLoS One. 2026 Jul 30;21(7):e0347719. doi: 10.1371/journal.pone.0347719 (PMC13423031; doi:10.1371/journal.pone.0347719)
Supplement: Attachment — Submitted filename: Response_to_Reviewers.docx [file pone.0347719.s007.docx]

**Response to Reviewers**

Manuscript ID: PONE-D-26-16353

*Bayesian hierarchical mixture modelling to derive probabilistic iELISA thresholds for bovine brucellosis in endemic dairy systems*

We thank the Academic Editor and both reviewers for their careful and constructive evaluation of our manuscript. We have addressed every editorial requirement and reviewer comment in full. Below we reproduce each point (in *italics*) and provide our response, followed by the corresponding revision. In the revised manuscript, all newly added or modified text is shown in **blue** to facilitate tracking. Line/section references refer to the revised file.

**A. Journal Requirements and Editorial Amendments**

**A1. PLOS ONE style and Vancouver referencing.** *Ensure file references, section headers, and the reference list conform to PLOS ONE / Vancouver style (sequential numbered brackets).*

**Response:** All in-text citations use sequential numbered brackets in order of first appearance, and the reference list has been checked for uniform Vancouver style (author list with *et al.* beyond six authors, article title, abbreviated journal name, year;volume:pages, with DOI where available). Section headings follow PLOS ONE structure.

**Changes in manuscript:** *Throughout; References section.*

**A2. Funding statement.** *Remove funding-related text from the manuscript; this belongs only in the online submission form.*

**Response:** The entire **Funding** subsection (“This research was funded by the Livestock and Dairy Development Project (LDDP)…”) has been deleted from the manuscript body. The funding information will be entered solely in the online submission form. The Acknowledgements section contains no funding text.

**Changes in manuscript:** *Declarations section — Funding paragraph deleted.*

**A3. Ethics statement placement.** *Ensure the ethics statement appears ONLY in the Methods section; delete it from any other section.*

**Response:** The ethics statement has been moved into the Materials and Methods section as a dedicated “Ethics statement” subsection, and the duplicate “Ethics approval” entry under Declarations has been removed. The statement (animal-use compliance, STARD-BLCM reporting, and AWEEC/BAU/2022/07 approval) now appears once, in Methods only.

**Changes in manuscript:** *Methods — new “Ethics statement” subsection; removed from Declarations.*

**A4. Missing Figure 4 and Table 4 callouts.** *Add in-text references to “Figure 4” and “Table 4” at contextually appropriate locations.*

**Response:** A “(Table 4)” callout has been added to the Population-Level Parameter Estimates paragraph where the weighted class prevalences are first reported, and a “(Figure 5; Table 6)” callout has been added to the Herd-Specific Analysis paragraph reporting herd-level true prevalence. Both figures/tables are now explicitly cited in the text.

**Changes in manuscript:** *Results — Population-Level Parameter Estimates (Table 4); Herd-Specific Analysis (Figure 4).*

**A5. Reference 16 (paratuberculosis) and reference chronology.** *Reference 16 discusses paratuberculosis (PTB) rather than brucellosis. Replace it with a highly relevant bovine brucellosis reference, re-check sequential numbering, and ensure uniform reference style.*

**Response:** We agree. Former reference 16 (Kibria et al., bovine *paratuberculosis* prevalence) was cited where the iELISA S/P% interpretation (negative ≤45%, doubtful 45–50%, positive >50%) is described — a brucellosis context. It has been replaced with a directly relevant bovine brucellosis serology reference: *Gall D, Nielsen K. Serological diagnosis of bovine brucellosis: a review of test performance and cost comparison. Rev Sci Tech. 2004;23:989–1002.* Because the replacement occupies the same citation-order position, all subsequent reference numbers (17–37) remain sequential and unchanged; we re-verified the full chronology and the uniformity of the reference style.

**Changes in manuscript:** *Methods (cut-off citation); Reference list, entry 16.*

**A6. Additional editorial items noted on the annotated PDF.** *Reviewer/editor margin notes: (i) use one consistent abbreviation for the model; (ii) define MCMC at first appearance; (iii) inconsistent italics on “Healthy < Latent < Diseased”; (iv) re-draft the prior-specification sentence.*

**Response:** (i) The model abbreviation is now uniformly **BHGMM** throughout (the heading “HB-GMM”, the Figure 1 caption “HGMM”, and the Supplementary File 2 caption have all been corrected). (ii) “Markov Chain Monte Carlo (MCMC)” is now spelled out at its first appearance (Data Processing) and abbreviated thereafter, with the later redundant definitions removed. (iii) The italic formatting of “Healthy < Latent < Diseased” has been normalised to roman text in both locations. (iv) The prior-specification sentence has been re-drafted to read: “…centred on moderate class separation (μ = 0.6, σ = 0.3). Variance parameters were assigned bounded Half-Normal hyperpriors…”

**Changes in manuscript:** *Methods (Data Processing; Ordered Means; Prior selection); Figure 1 caption; Supplementary File 2 caption.*

**B. Reviewer 1**

**B1. Vaccination status and the intermediate (latent) group.** *Were vaccinated animals included? Clarify how vaccination status affects interpretation of the intermediate/uncertain group.*

**Response:** None of the sampled herds practised brucellosis vaccination. In Bangladesh, systematic mass vaccination with strain 19 (S19) or RB51 is not implemented and is generally restricted at the national level. Consequently, the observed antibody (S/P%) distributions reflect *natural infection kinetics* rather than vaccine-induced seroreactivity. This materially simplifies interpretation of the intermediate group: the latent component cannot be attributed to vaccine antibodies and is therefore best interpreted as genuinely infected animals with subclinical/persistent responses, strengthening the epidemiological meaning of the dual-cutoff “gray zone.” We have added an explicit statement in Methods and reinforced the interpretation in the Discussion.

**Changes in manuscript:** *Methods — Study design, target and study population; Discussion (latent-class interpretation).*

**B2. External validation and comparison with other latent-class approaches.** *Add a detailed paragraph on plans for external validation of the BHGMM across geographical regions and on how it compares with other latent-class approaches (standard LCA, traditional cut-offs).*

**Response:** A dedicated paragraph has been added to the Discussion describing (a) prospective external validation by re-estimating and validating the BHGMM in independent endemic populations — e.g., other South Asian and endemic dairy systems that differ in infection pressure, breed, and management — to test transportability of the dual-cutoff structure; and (b) head-to-head benchmarking against conventional latent class analysis (LCA) against an imperfect reference, multi-test Bayesian latent class models, and fixed manufacturer cut-offs, noting that the mixture formulation preserves the continuous antibody signal and explicitly recovers the latent component, ideally with partial bacteriological/molecular confirmation.

**Changes in manuscript:** *Discussion — new paragraph preceding the limitations.*

**C. Reviewer 2**

**C1. Readability — stepwise methodology / flowchart.** *Provide a clear, sequential, step-by-step workflow summarising the diagnostic testing pipeline, sample collection, and the rationale for choosing the BHGMM over fixed binary cut-offs, accessible to non-experts.*

**Response:** We have added a new Methods subsection, “Diagnostic and analytical workflow,” containing an eight-step text/ASCII flowchart (field sampling → handling → iELISA → transformation → component selection → BHGMM fitting → threshold derivation → validation/output) followed by a plain-language paragraph explaining why a mixture model with herd-specific random effects was preferred to a single fixed cut-off (it preserves the continuous, graded antibody signal, recovers the latent “gray-zone” component, derives herd-adapted dual thresholds, and propagates diagnostic uncertainty).

**Changes in manuscript:** *Methods — new “Diagnostic and analytical workflow” subsection.*

**C2. Paired milk sampling at 21-day intervals.** *Testing paired milk samples taken 21 days apart would capture antibody titre fluctuations.*

**Response:** We agree this is a valuable longitudinal design, but it was outside the scope of this study. This was a *cross-sectional* study designed to characterise population-level antibody distributions across 2,696 samples; repeated paired sampling 21 days apart was logistically and financially unfeasible under field conditions. Importantly, the BHGMM is specifically constructed to extract population heterogeneity and diagnostic uncertainty from a *single* cross-sectional measurement by modelling the full continuous distribution as a mixture of latent subpopulations, so individual-level longitudinal re-testing is not required to estimate the thresholds and prevalences. We have added this explicitly to the Limitations.

**Changes in manuscript:** *Discussion — Limitations paragraph.*

**C3. Farm-level risk factors.** *Expand the Discussion on farm-level risk factors and their influence on the continuous antibody distribution.*

**Response:** A new Discussion paragraph links the pronounced between-herd variation in true prevalence (≈1–67%) and confirmatory cut-offs to plausible farm-level drivers — herd size, stocking density, replacement/movement of animals, biosecurity, calving and abortion management, and trade practices. We explain how higher infection pressure shifts the mixture weights toward the latent and diseased components and raises herd-specific thresholds, how these effects are absorbed by the herd random effects, and how incorporating such covariates as herd-level predictors of the mixing proportions in future models would move the framework from describing heterogeneity to explaining it for risk-based management.

**Changes in manuscript:** *Discussion — new farm-level risk-factor paragraph.*

*We believe these revisions have substantially strengthened the manuscript and addressed all concerns. We thank the editor and reviewers again for their time and constructive input.*
