# Supplementary material for: Bayesian hierarchical mixture modelling to derive probabilistic iELISA thresholds for bovine brucellosis in endemic dairy systems
Source: PLoS One. 2026 Jul 30;21(7):e0347719. doi: 10.1371/journal.pone.0347719 (PMC13423031; doi:10.1371/journal.pone.0347719)
Supplement: Peer review history

# Peer review: Decision Letter 0

**DOI:** 10.1371/journal.pone.0347719.r001

**Authors:** Saurabh Gupta

23 Jun 2026

PONE-D-26-16353

Bayesian hierarchical mixture modelling to derive probabilistic iELISA thresholds for bovine brucellosis in endemic dairy systems

PLOS One

Dear Dr. Rahman,

Thank you for submitting your manuscript to PLOS ONE. After careful consideration, we feel that it has merit but does not fully meet PLOS ONE’s publication criteria as it currently stands. Therefore, we invite you to submit a revised version of the manuscript that addresses the points raised during the review process.

==============================

**ACADEMIC EDITOR:**

Please find attached the reviewer comments for your manuscript. Based on the review process, the paper requires **major revision**  before it can be considered further.

Kindly revise the manuscript carefully in accordance with all reviewer comments and suggestions.

==============================

Please submit your revised manuscript by Aug 07 2026 11:59PM. If you will need more time than this to complete your revisions, please reply to this message or contact the journal office at plosone@plos.org. When you're ready to submit your revision, log on to https://www.editorialmanager.com/pone/ and select the 'Submissions Needing Revision' folder to locate your manuscript file.

Please include the following items when submitting your revised manuscript:

- A letter that responds to each point raised by the academic editor and reviewer(s). You should upload this letter as a separate file labeled 'Response to Reviewers'.
- A marked-up copy of your manuscript that highlights changes made to the original version. You should upload this as a separate file labeled 'Revised Manuscript with Track Changes'.
- An unmarked version of your revised paper without tracked changes. You should upload this as a separate file labeled 'Manuscript'.

If you would like to make changes to your financial disclosure, please include your updated statement in your cover letter. Guidelines for resubmitting your figure files are available below the reviewer comments at the end of this letter.

If applicable, we recommend that you deposit your laboratory protocols in protocols.io to enhance the reproducibility of your results. Protocols.io assigns your protocol its own identifier (DOI) so that it can be cited independently in the future. For instructions see: https://journals.plos.org/plosone/s/submission-guidelines#loc-laboratory-protocols. Additionally, PLOS ONE offers an option for publishing peer-reviewed Lab Protocol articles, which describe protocols hosted on protocols.io. Read more information on sharing protocols at https://plos.org/protocols?utm_medium=editorial-email&utm_source=authorletters&utm_campaign=protocols.

As the corresponding author, your ORCID iD is verified in the submission system and will appear in the published article. PLOS supports the use of ORCID, and we encourage all coauthors to register for an ORCID iD and use it as well. Please encourage your coauthors to verify their ORCID iD within the submission system before final acceptance, as unverified ORCID iDs will not appear in the published article. *Only*  the individual author can complete the verification step; PLOS staff *cannot*  verify ORCID iDs on behalf of authors.

We look forward to receiving your revised manuscript.

Kind regards,

Saurabh Gupta, Ph.D.

Academic Editor

PLOS One

Journal Requirements:

When submitting your revision, we need you to address these additional requirements.

1. Please ensure that your manuscript meets PLOS ONE's style requirements, including those for file naming. The PLOS ONE style templates can be found at

https://journals.plos.org/plosone/s/file?id=wjVg/PLOSOne_formatting_sample_main_body.pdf and

https://journals.plos.org/plosone/s/file?id=ba62/PLOSOne_formatting_sample_title_authors_affiliations.pdf.

2. Please be informed that funding information should not appear in the Acknowledgments section or other areas of your manuscript. We will only publish funding information present in the Funding Statement section of the online submission form. Please remove any funding-related text from the manuscript.

3. Your ethics statement should only appear in the Methods section of your manuscript. If your ethics statement is written in any section besides the Methods, please move it to the Methods section and delete it from any other section. Please ensure that your ethics statement is included in your manuscript, as the ethics statement entered into the online submission form will not be published alongside your manuscript.

4. Please ensure that you refer to Figure 4 in your text as, if accepted, production will need this reference to link the reader to the figure.

5. We note you have included a table to which you do not refer in the text of your manuscript. Please ensure that you refer to Table 4 in your text; if accepted, production will need this reference to link the reader to the Table.

6. If the reviewer comments include a recommendation to cite specific previously published works, please review and evaluate these publications to determine whether they are relevant and should be cited. There is no requirement to cite these works unless the editor has indicated otherwise.

Additional Editor Comments (if provided):

Dear Author,

Please find attached the reviewer comments for your manuscript. Based on the review process, the paper requires major revision before it can be considered further.

Kindly revise the manuscript carefully in accordance with all reviewer comments and suggestions.

We look forward to receiving your revised submission.

[Note: HTML markup is below. Please do not edit.]

Reviewers' comments:

Reviewer's Responses to Questions

**Comments to the Author**

1. Is the manuscript technically sound, and do the data support the conclusions?

The manuscript must describe a technically sound piece of scientific research with data that supports the conclusions. Experiments must have been conducted rigorously, with appropriate controls, replication, and sample sizes. The conclusions must be drawn appropriately based on the data presented.

Reviewer #1: Yes

Reviewer #2: Yes

**********

2. Has the statistical analysis been performed appropriately and rigorously?

Reviewer #1: Yes

Reviewer #2: Yes

**********

3. Have the authors made all data underlying the findings in their manuscript fully available?

The PLOS Data policy requires authors to make all data underlying the findings described in their manuscript fully available without restriction, with rare exception (please refer to the Data Availability Statement in the manuscript PDF file). The data should be provided as part of the manuscript or its supporting information, or deposited to a public repository. For example, in addition to summary statistics, the data points behind means, medians and variance measures should be available. If there are restrictions on publicly sharing data—e.g. participant privacy or use of data from a third party—those must be specified.

Reviewer #1: Yes

Reviewer #2: Yes

**********

4. Is the manuscript presented in an intelligible fashion and written in standard English?

PLOS ONE does not copyedit accepted manuscripts, so the language in submitted articles must be clear, correct, and unambiguous. Any typographical or grammatical errors should be corrected at revision, so please note any specific errors here.

Reviewer #1: Yes

Reviewer #2: No

**********

5. Review Comments to the Author

Please use the space provided to explain your answers to the questions above. You may also include additional comments for the author, including concerns about dual publication, research ethics, or publication ethics. (Please upload your review as an attachment if it exceeds 20,000 characters)

Reviewer #1: This is a well-designed and methodologically strong study addressing an important challenge in bovine brucellosis diagnostics under endemic conditions.It is a innovative and well suited to endemic settings where binary cut-offs are often inadequate. Only minor revisions are required to improve biological interpretation of the study, and strengthen discussion of limitations and practical implementation.

Since vaccination can influence antibody titres it would be benefitial to discuss whether vaccinated animals were included and how vaccination may affect interpretation of the intermediate serological group. Also, the manuscript would be strengthened by discussing plans for external validation in different geographical regions and the compaison with external approaches.

Reviewer #2: Bayesian hierarchical mixture modelling to derive probabilistic iELISA thresholds for bovine brucellosis in endemic dairy systems ...Its a very unique work but not many will understand the basics of various tests and methodologies adopted and why. ....Please write in flow chart or stepwise manner why you have opted for the particulat method ...All methodologie in one page would have been better. Collection and testing of around 2700 milk samples from different herds were very good but have you tested the another set of milk samples from 21 days apart from the same animal would have been valuable to come to the conclusion because of high or low antibody titre in milk. At least few hundred paired milk samples would have been great. Style of references has to be uniform all around.Why reference 16 is on PTB and not Brucellosis. Few reference details are missing or not uniform in style. Risk factors across the farm and their influences and also advantages and limitations of the study is inadequately elaborated. One more comprehensive reading and makeshift of the manuscript is suggested so that those who are not expert in this area can rather understand your study precisely.

**********

6. PLOS authors have the option to publish the peer review history of their article (what does this mean?). If published, this will include your full peer review and any attached files.

If you choose “no”, your identity will remain anonymous but your review may still be made public.

**Do you want your identity to be public for this peer review?**  For information about this choice, including consent withdrawal, please see our Privacy Policy.

Reviewer #1: No

Reviewer #2: No

**********

[NOTE: If reviewer comments were submitted as an attachment file, they will be attached to this email and accessible via the submission site. Please log into your account, locate the manuscript record, and check for the action link "View Attachments". If this link does not appear, there are no attachment files.]

To ensure your figures meet our technical requirements, please review our figure guidelines: https://journals.plos.org/plosone/s/figures

You may also use PLOS’s free figure tool, NAAS, to help you prepare publication quality figures: https://journals.plos.org/plosone/s/figures#loc-tools-for-figure-preparation.

NAAS will assess whether your figures meet our technical requirements by comparing each figure against our figure specifications.

Attachment Submitted filename: PLOS One IMG_20260518_0001.pdf

# Author comment: Author response to Decision Letter 1

**DOI:** 10.1371/journal.pone.0347719.r002

29 Jun 2026

Response to Reviewers

Manuscript ID: PONE-D-26-16353

Bayesian hierarchical mixture modelling to derive probabilistic iELISA thresholds for bovine brucellosis in endemic dairy systems

We thank the Academic Editor and both reviewers for their careful and constructive evaluation of our manuscript. We have addressed every editorial requirement and reviewer comment in full. Below we reproduce each point (in italics) and provide our response, followed by the corresponding revision. In the revised manuscript, all newly added or modified text is shown in blue to facilitate tracking. Line/section references refer to the revised file.

A. Journal Requirements and Editorial Amendments

A1. PLOS ONE style and Vancouver referencing. Ensure file references, section headers, and the reference list conform to PLOS ONE / Vancouver style (sequential numbered brackets).

Response: All in-text citations use sequential numbered brackets in order of first appearance, and the reference list has been checked for uniform Vancouver style (author list with et al. beyond six authors, article title, abbreviated journal name, year;volume:pages, with DOI where available). Section headings follow PLOS ONE structure.

Changes in manuscript: Throughout; References section.

A2. Funding statement. Remove funding-related text from the manuscript; this belongs only in the online submission form.

Response: The entire Funding subsection (“This research was funded by the Livestock and Dairy Development Project (LDDP)…”) has been deleted from the manuscript body. The funding information will be entered solely in the online submission form. The Acknowledgements section contains no funding text.

Changes in manuscript: Declarations section — Funding paragraph deleted.

A3. Ethics statement placement. Ensure the ethics statement appears ONLY in the Methods section; delete it from any other section.

Response: The ethics statement has been moved into the Materials and Methods section as a dedicated “Ethics statement” subsection, and the duplicate “Ethics approval” entry under Declarations has been removed. The statement (animal-use compliance, STARD-BLCM reporting, and AWEEC/BAU/2022/07 approval) now appears once, in Methods only.

Changes in manuscript: Methods — new “Ethics statement” subsection; removed from Declarations.

A4. Missing Figure 4 and Table 4 callouts. Add in-text references to “Figure 4” and “Table 4” at contextually appropriate locations.

Response: A “(Table 4)” callout has been added to the Population-Level Parameter Estimates paragraph where the weighted class prevalences are first reported, and a “(Figure 5; Table 6)” callout has been added to the Herd-Specific Analysis paragraph reporting herd-level true prevalence. Both figures/tables are now explicitly cited in the text.

Changes in manuscript: Results — Population-Level Parameter Estimates (Table 4); Herd-Specific Analysis (Figure 4).

A5. Reference 16 (paratuberculosis) and reference chronology. Reference 16 discusses paratuberculosis (PTB) rather than brucellosis. Replace it with a highly relevant bovine brucellosis reference, re-check sequential numbering, and ensure uniform reference style.

Response: We agree. Former reference 16 (Kibria et al., bovine paratuberculosis prevalence) was cited where the iELISA S/P% interpretation (negative ≤45%, doubtful 45–50%, positive >50%) is described — a brucellosis context. It has been replaced with a directly relevant bovine brucellosis serology reference: Gall D, Nielsen K. Serological diagnosis of bovine brucellosis: a review of test performance and cost comparison. Rev Sci Tech. 2004;23:989–1002. Because the replacement occupies the same citation-order position, all subsequent reference numbers (17–37) remain sequential and unchanged; we re-verified the full chronology and the uniformity of the reference style.

Changes in manuscript: Methods (cut-off citation); Reference list, entry 16.

A6. Additional editorial items noted on the annotated PDF. Reviewer/editor margin notes: (i) use one consistent abbreviation for the model; (ii) define MCMC at first appearance; (iii) inconsistent italics on “Healthy < Latent < Diseased”; (iv) re-draft the prior-specification sentence.

Response: (i) The model abbreviation is now uniformly BHGMM throughout (the heading “HB-GMM”, the Figure 1 caption “HGMM”, and the Supplementary File 2 caption have all been corrected). (ii) “Markov Chain Monte Carlo (MCMC)” is now spelled out at its first appearance (Data Processing) and abbreviated thereafter, with the later redundant definitions removed. (iii) The italic formatting of “Healthy < Latent < Diseased” has been normalised to roman text in both locations. (iv) The prior-specification sentence has been re-drafted to read: “…centred on moderate class separation (μ = 0.6, σ = 0.3). Variance parameters were assigned bounded Half-Normal hyperpriors…”

Changes in manuscript: Methods (Data Processing; Ordered Means; Prior selection); Figure 1 caption; Supplementary File 2 caption.

B. Reviewer 1

B1. Vaccination status and the intermediate (latent) group. Were vaccinated animals included? Clarify how vaccination status affects interpretation of the intermediate/uncertain group.

Response: None of the sampled herds practised brucellosis vaccination. In Bangladesh, systematic mass vaccination with strain 19 (S19) or RB51 is not implemented and is generally restricted at the national level. Consequently, the observed antibody (S/P%) distributions reflect natural infection kinetics rather than vaccine-induced seroreactivity. This materially simplifies interpretation of the intermediate group: the latent component cannot be attributed to vaccine antibodies and is therefore best interpreted as genuinely infected animals with subclinical/persistent responses, strengthening the epidemiological meaning of the dual-cutoff “gray zone.” We have added an explicit statement in Methods and reinforced the interpretation in the Discussion.

Changes in manuscript: Methods — Study design, target and study population; Discussion (latent-class interpretation).

B2. External validation and comparison with other latent-class approaches. Add a detailed paragraph on plans for external validation of the BHGMM across geographical regions and on how it compares with other latent-class approaches (standard LCA, traditional cut-offs).

Response: A dedicated paragraph has been added to the Discussion describing (a) prospective external validation by re-estimating and validating the BHGMM in independent endemic populations — e.g., other South Asian and endemic dairy systems that differ in infection pressure, breed, and management — to test transportability of the dual-cutoff structure; and (b) head-to-head benchmarking against conventional latent class analysis (LCA) against an imperfect reference, multi-test Bayesian latent class models, and fixed manufacturer cut-offs, noting that the mixture formulation preserves the continuous antibody signal and explicitly recovers the latent component, ideally with partial bacteriological/molecular confirmation.

Changes in manuscript: Discussion — new paragraph preceding the limitations.

C. Reviewer 2

C1. Readability — stepwise methodology / flowchart. Provide a clear, sequential, step-by-step workflow summarising the diagnostic testing pipeline, sample collection, and the rationale for choosing the BHGMM over fixed binary cut-offs, accessible to non-experts.

Response: We have added a new Methods subsection, “Diagnostic and analytical workflow,” containing an eight-step text/ASCII flowchart (field sampling → handling → iELISA → transformation → component selection → BHGMM fitting → threshold derivation → validation/output) followed by a plain-language paragraph explaining why a mixture model with herd-specific random effects was preferred to a single fixed cut-off (it preserves the continuous, graded antibody signal, recovers the latent “gray-zone” component, derives herd-adapted dual thresholds, and propagates diagnostic uncertainty).

Changes in manuscript: Methods — new “Diagnostic and analytical workflow” subsection.

C2. Paired milk sampling at 21-day intervals. Testing paired milk samples taken 21 days apart would capture antibody titre fluctuations.

Response: We agree this is a valuable longitudinal design, but it was outside the scope of this study. This was a cross-sectional study designed to characterise population-level antibody distributions across 2,696 samples; repeated paired sampling 21 days apart was logistically and financially unfeasible under field conditions. Importantly, the BHGMM is specifically constructed to extract population heterogeneity and diagnostic uncertainty from a single cross-sectional measurement by modelling the full continuous distribution as a mixture of latent subpopulations, so individual-level longitudinal re-testing is not required to estimate the thresholds and prevalences. We have added this explicitly to the Limitations.

Changes in manuscript: Discussion — Limitations paragraph.

C3. Farm-level risk factors. Expand the Discussion on farm-level risk factors and their influence on the continuous antibody distribution.

Response: A new Discussion paragraph links the pronounced between-herd variation in true prevalence (≈1–67%) and confirmatory cut-offs to plausible farm-level drivers — herd size, stocking density, replacement/movement of animals, biosecurity, calving and abortion management, and trade practices. We explain how higher infection pressure shifts the mixture weights toward the latent and diseased components and raises herd-specific thresholds, how these effects are absorbed by the herd random effects, and how incorporating such covariates as herd-level predictors of the mixing proportions in future models would move the framework from describing heterogeneity to explaining it for risk-based management.

Changes in manuscript: Discussion — new farm-level risk-factor paragraph.

We believe these revisions have substantially strengthened the manuscript and addressed all concerns. We thank the editor and reviewers again for their time and constructive input.

Attachment Submitted filename: Response_to_Reviewers.docx

# Editor report: Decision Letter 1

**DOI:** 10.1371/journal.pone.0347719.r003

**Authors:** Saurabh Gupta

14 Jul 2026

<p>Bayesian hierarchical mixture modelling to derive probabilistic iELISA thresholds for bovine brucellosis in endemic dairy systems

PONE-D-26-16353R1

Dear Dr. -->Rahman,

We’re pleased to inform you that your manuscript has been judged scientifically suitable for publication and will be formally accepted for publication once it meets all outstanding technical requirements.

Within one week, you’ll receive an e-mail detailing the required amendments. When these have been addressed, you’ll receive a formal acceptance letter and your manuscript will be scheduled for publication.

An invoice will be generated when your article is formally accepted. Please note, if your institution has a publishing partnership with PLOS and your article meets the relevant criteria, all or part of your publication costs will be covered. Please make sure your user information is up-to-date by logging into Editorial Manager at Editorial Manager® and clicking the ‘Update My Information' link at the top of the page. For questions related to billing, please contact billing support.

If your institution or institutions have a press office, please notify them about your upcoming paper to help maximize its impact. If they’ll be preparing press materials, please inform our press team as soon as possible -- no later than 48 hours after receiving the formal acceptance. Your manuscript will remain under strict press embargo until 2 pm Eastern Time on the date of publication. For more information, please contact onepress@plos.org.

Kind regards,

Saurabh Gupta, Ph.D.

Academic Editor

PLOS One

Additional Editor Comments (optional):

Reviewers' comments:

# Editor report: Acceptance letter

**DOI:** 10.1371/journal.pone.0347719.r004

**Authors:** Saurabh Gupta

PONE-D-26-16353R1

PLOS One

Dear Dr. Rahman,

I'm pleased to inform you that your manuscript has been deemed suitable for publication in PLOS One. Congratulations! Your manuscript is now being handed over to our production team.

At this stage, our production department will prepare your paper for publication. This includes ensuring the following:

* All references, tables, and figures are properly cited

* All relevant supporting information is included in the manuscript submission,

* There are no issues that prevent the paper from being properly typeset

You will receive further instructions from the production team, including instructions on how to review your proof when it is ready. Please keep in mind that we are working through a large volume of accepted articles, so please give us a few days to review your paper and let you know the next and final steps.

Lastly, if your institution or institutions have a press office, please let them know about your upcoming paper now to help maximize its impact. If they'll be preparing press materials, please inform our press team within the next 48 hours. Your manuscript will remain under strict press embargo until 2 pm Eastern Time on the date of publication. For more information, please contact onepress@plos.org.

You will receive an invoice from PLOS for your publication fee after your manuscript has reached the completed accept phase. If you receive an email requesting payment before acceptance or for any other service, this may be a phishing scheme. Learn how to identify phishing emails and protect your accounts at https://explore.plos.org/phishing.

If we can help with anything else, please email us at customercare@plos.org.

Thank you for submitting your work to PLOS ONE and supporting open access.

Kind regards,

PLOS ONE Editorial Office Staff

on behalf of

Dr. Saurabh Gupta

Academic Editor

PLOS One
